# Obesity and Environmental Risk Factors Significantly Modify the Association between Ischemic Stroke and the Hero Chaperone *C19orf53*

**DOI:** 10.3390/life14091158

**Published:** 2024-09-12

**Authors:** Irina Shilenok, Ksenia Kobzeva, Alexey Deykin, Vladimir Pokrovsky, Evgeny Patrakhanov, Olga Bushueva

**Affiliations:** 1 Laboratory of Genomic Research, Research Institute for Genetic and Molecular Epidemiology, Kursk State Medical University, 305041 Kursk, Russia; 2 Division of Neurology, Kursk Emergency Hospital, 305035 Kursk, Russia; 3 Laboratory of Genome Editing for Biomedicine and Animal Health, Belgorod State National Research University, 308015 Belgorod, Russia; 4 Department of Pharmacology and Clinical Pharmacology, Belgorod State National Research University, 308015 Belgorod, Russia; 5 Laboratory of Genetic Technologies and Gene Editing for Biomedicine and Veterinary Medicine, Belgorod State National Research University, 308015 Belgorod, Russia; 6 Department of Biology, Medical Genetics and Ecology, Kursk State Medical University, 305041 Kursk, Russia

**Keywords:** C19orf53, chromosome 19 open reading frame 53, SNP, obesity, low fruit and vegetable intake, low physical activity, chaperones, heat-resistant obscure, hero, ischemic stroke

## Abstract

The unique chaperone-like properties of C19orf53, discovered in 2020 as a “hero” protein, make it an intriguing subject for research in relation to ischemic stroke (IS). Our pilot study aimed to investigate whether C19orf53 SNPs are associated with IS. DNA samples from 2138 Russian subjects (947 IS and 1308 controls) were genotyped for 7 *C19orf53* SNPs using probe-based PCR. Dominant (D), recessive (R), and log-additive (A) regression models in relation to the effect alleles (EA) were used to interpret associations. An increased risk of IS was associated with rs10104 (EA G; P_bonf(R)_ = 0.0009; P_bonf(A)_ = 0.0004), rs11666524 (EA A; P_bonf(R)_ = 0.003; P_bonf(A)_ = 0.02), rs346158 (EA C; P_bonf(R)_ = 0.006; P_bonf(A)_ = 0.045), and rs2277947 (EA A; P_bonf(R)_ = 0.002; P_bonf(A)_ = 0.01) in patients with obesity; with rs11666524 (EA A; P_bonf(R)_ = 0.02), rs346157 (EA G; P_bonf(R)_ = 0.036), rs346158 (EA C; P_bonf(R)_ = 0.005), and rs2277947 (EA A; P_bonf(R)_ = 0.02) in patients with low fruit and vegetable intake; and with rs10104 (EA G; P_bonf(R)_ = 0.03) and rs11666524 (EA A; P_bonf(R)_ = 0.048) in patients with low physical activity. In conclusion, our pilot study provides comprehensive genetic and bioinformatic evidence of the involvement of *C19orf53* in IS risk.

## 1. Introduction

An ischemic stroke (IS) is recognized as a major contributor to morbidity and mortality worldwide [1]. An IS is a localized brain injury resulting from a substantial decrease in cerebral blood flow within major cerebral arteries caused by embolism or thrombosis usually following atherosclerotic plaque rapture [2]. Acute ischemia leads to cell death directly via severe metabolic deficits or via secondary alteration as a result of inflammation and blood–brain barrier opening [3].

A crucial mechanism in minimizing cellular damage in an ischemia–reperfusion injury involves the utilization of chaperone machinery [4,5]. Chaperones form a functionally linked network of proteins dedicated to ensuring the quality of proteome—a pivotal process for cellular protection against lethal stress [6,7]. Moreover, chaperones have been implicated in a wide range of processes markedly important for IS etiology, such as vascular tone regulation [8,9,10], atherosclerotic plaque formation and dynamics [11,12,13], and platelet aggregation [14,15].

C19orf53, also known as Chromosome 19 Open Reading Frame 53, plays a role in mediating excessive cell proliferation, leading to metabolic imbalance and increased oxidative stress in response to toxins [16]. Associated with various biological pathways, including rRNA biogenesis, ovarian cancer, and Zika virus replication [17,18], C19orf53 gained attention in 2020 when Tsuboyama K et al. identified it along with other small heat-resistant proteins displaying chaperone-like activity in cell culture lysate [19]. Referred to as “heat-resistant obscure” or “Hero”, C19orf53 exhibited resilience, emphasizing its chaperone-like characteristics. Given the significance of chaperones in IS, exploring the newfound properties of C19orf53 protein is crucial for understanding its potential implications in the disease. All Hero genes previously studied by us showed significant associations with the risk of developing an IS [20,21,22], suggesting their potentially significant role in the molecular mechanisms of IS development, most likely due to their pronounced chaperone-like properties. In addition, a recent study has established the role of C19orf53 in suppressing the pathological aggregation of TAR DNA-binding protein 43, which plays an important role in neurodegenerative diseases [23]. Given the significance of chaperones in IS, exploring the newfound properties of C19orf53 protein is crucial for understanding its potential implications in the disease.

The aim of our study was to investigate whether the polymorphism of the *C19orf53* gene, encoding a heat-resistant obscure (Hero) protein with chaperone-like activity, is associated with the risk and clinical features of ischemic stroke.

## 2. Materials and Methods

**Figure 1:**
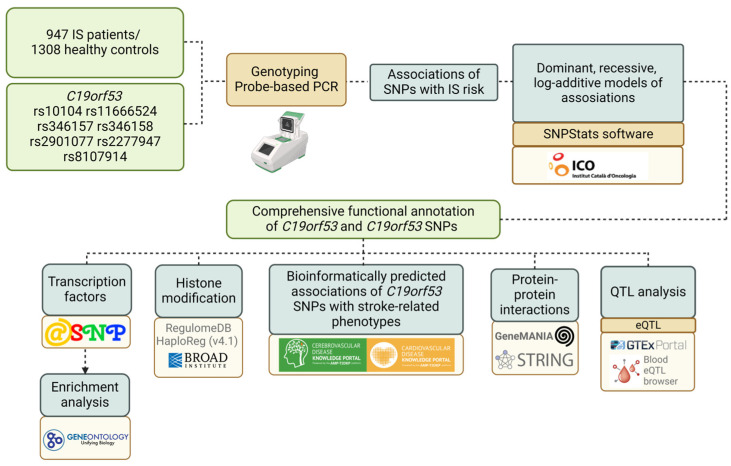
The outline of the study design.

The outline of the study design is shown in Figure 1.

### 2.1. Experimental Design

The study included 2138 unrelated individuals from central Russia, comprising 947 IS patients and 1308 healthy individuals. The Ethical Review Committee of Kursk State Medical University approved the study protocol, and all participants provided written informed consent. Inclusion criteria for the study required participants to have self-declared Russian ancestry and to be born in central Russia.

Table 1 provides general clinical characteristics of IS patients and controls.

The patients were enrolled in the study during two distinct periods: from 2015 to 2017 at the Regional Vascular Center of Kursk Regional Clinical Hospital [24,25] and from 2010 to 2012 at the Neurology Clinics of Kursk Emergency Medicine Hospital [26,27]. Inclusion criteria for the study required patients to have a diagnosis of ischemic stroke, confirmed by neurologists during the acute phase of the disease based on clinical and neurological evaluations and verified by computed tomography or magnetic resonance imaging of the brain performed within the first 48 hours following the stroke event. The study included the following subtypes of ischemic stroke: atherothrombotic, cardioembolic, and stroke of unknown etiology. Exclusion criteria included patients with liver or kidney failure, endocrine, autoimmune, oncological, or other conditions that could lead to an acute cerebrovascular accident. Additionally, patients with hemorrhagic stroke, secondary intracerebral hemorrhage, hemodynamic, hemorheological, or dissection stroke as well as those with traumatic brain injury were excluded. All included IS patients had a documented history of hypertension and were receiving antihypertensive treatment.

The control group consisted of healthy volunteers who had normal blood pressure, did not receive antihypertensive therapy, and showed no clinical symptoms of cardiovascular, cerebrovascular, or other significant illnesses. Healthy individuals were included in the control group if their systolic blood pressure was below 130 mm Hg and diastolic blood pressure was below 85 mm Hg, based on at least three separate measurements. Controls from the Kursk region were selected from hospitals during routine medical examinations conducted in public institutions and industrial establishments. This group was recruited from the same population and during the same time period.

In accordance with WHO guidelines, low fruit and vegetable consumption was defined as consuming less than 400 g per day. Adequate consumption of fresh vegetables and fruits was defined as consuming 400 g or more, equivalent to 3–4 servings per day, excluding starchy tubers like potatoes.

The following criteria were used to choose the SNPs: have a minor allele frequency of at least 0.05 in the European population and be distinguished by a high regulatory potential.

The bioinformatic tools LD TAG SNP Selection (TagSNP) and SNPinfo Web Server (https://snpinfo.niehs.nih.gov/ (accessed on 15 March 2022)) were utilized to choose SNPs based on the reference haplotypic structure of the Caucasian population (CEU) of the project HapMap. The *C19orf53* (Chromosome 19 Open Reading Frame 53, ID: 26135) gene contains seven SNPs (rs10104, rs11666524, rs346157, rs346158, rs2901077, rs2277947, and rs8107914). SNP rs10104 is a missense variant, SNPs rs11666524, rs346157, rs346158, rs2901077 are located in introns, and SNPs rs2277947 and rs8107914 are located in the 3 prime UTRs.

### 2.2. Genetic Analysis

Genotyping was carried out by the Laboratory of Genomic Research at the Research Institute for Genetic and Molecular Epidemiology. Venous blood samples (up to 5 mL) were drawn from each participant’s cubital vein and placed in EDTA-coated tubes, which were then stored at −20 °C until processing. Genomic DNA was extracted from the thawed blood samples using standard phenol/chloroform extraction and ethanol precipitation methods. The purity, quality, and concentration of the extracted DNA were evaluated using a NanoDrop spectrophotometer (Thermo Fisher Scientific, Waltham, MA, USA).

Detection of SNP variations in the *C19orf53* gene was conducted using Taq-Man-based PCR following protocols developed at the Research Institute for Genetic and Molecular Epidemiology. Primer design was carried out using Primer3 software version 4.1.0 [28], with the specific primers and probes listed in Table S1. The real-time PCR was performed in a 25 µL reaction mixture containing 1.5 units of Hot Start Taq DNA polymerase (Biolabmix, Novosibirsk, Russia), approximately 10 ng of DNA, and the following reagent concentrations: 0.25 μM of each primer; 0.1 μM of each probe; 250 μM of each dNTP; 2.5 mM MgCl_2_; 1xPCR buffer (67 mM Tris-HCl, pH 8.8, 16.6 mM (NH_4_)_2_SO_4_, 0.01% Tween-20). The PCR procedure comprised an initial denaturation for 10 min at 95 °C, followed by 39 cycles of 92 °C for 30 s and 53 °C, 57.5 °C, 59 °C, 61 °C, 63 °C, and 65 °C for 1 min (for rs346158, rs8107914, rs2901077 and rs11666524, rs10104, rs346157, and rs2277947, respectively). Supplementary Figure S1 shows allelic discrimination plots for *C19orf53* assays designed for this study. Ten percent of the DNA samples were genotyped twice, blinded to the case-control status, in order to assure quality control. Over 99% of the data were concordant.

### 2.3. Statistical and Bioinformatic Analysis

Statistical power was calculated prior to the study using genetic association study power calculator [29]. Association analysis between the *C19orf53* gene polymorphisms and IS risk could detect the genotype relative risk of 1.2–1.4, assuming 0.80 power and 5% type I error (α = 0.05) on the sample size of 947 cases and 1308 controls.

The STATISTICA software (v13.3, Santa Clara, California, USA) was utilized for statistical processing. The normality of the distribution for quantitative data was assessed using the Shapiro–Wilk’s test. Given that the majority of quantitative parameters exhibited deviations from normal distribution, they were presented as the median (Me) along with the first and third quartiles [Q1 and Q3]. To compare quantitative variables among two independent groups, the Mann–Whitney test was performed. For categorical variables, differences in statistical significance were evaluated using Pearson’s chi-squared test.

The genotype distributions were assessed for compliance with Hardy–Weinberg equilibrium using Fisher’s exact test. The study groups’ genotype frequencies and their associations with disease risk were analyzed using SNPStats software (https://www.snpstats.net/start.htm) (accessed on 23 April 2024). Additive, dominant and recessive models were considered for the genotype association analysis. All calculations were made in relation to the effect allele (EA). The Bonferroni correction was applied additionally to account for multiple comparisons. Associations within the entire group of IS patients/controls were adjusted for age, gender, and smoking status. When information regarding the environmental risk factor was unavailable in the control group, associations were analyzed by comparing the presence or absence of the risk factor in the patient group to the overall control group.

The functional effects of *C19orf53* SNPs were analyzed using the following bioinformatics resources:

- The GTExportal (http://www.gtexportal.org/ (accessed on 14 May 2024)) was employed to analyze the expression levels of the studied genes in the brain, whole blood, and blood vessels as well as to examine the expression quantitative trait loci (eQTLs) [30].
- For the examination of *C19orf53* SNPs binding to quantitative expression trait loci (eQTL) in peripheral blood, the eQTLGen resource available at https://www.eqtlgen.org/ (accessed on 14 May 2024) was employed [31].
- HaploReg (v4.2), a bioinformatics tool available at https://pubs.broadinstitute.org/mammals/haploreg/haploreg.php (accessed on 14 May 2024), was utilized to assess the associations between *C19orf53* SNPs and specific histone modifications indicative of promoters and enhancers. These modifications included acetylation of lysine residues at positions 27 and 9 of the histone H3 protein as well as mono-methylation at position 4 (H3K4me1) and tri-methylation at position 4 (H3K4me3) of the histone H3 protein. Additionally, the tool was applied to investigate the positioning of SNPs in DNase hypersensitive regions, regulatory motif sites, and locations binding to regulatory proteins [32].
- Bioinformatic tool STRING database (https://string-db.org/ (accessed on 14 May 2024)) was employed to analyze the primary interaction partners of C19orf53. Additionally, the analysis of biological processes and molecular functions related to interactions with key functionally related proteins was conducted using the STRING database [33].
- The mechanisms of interactions between *C19orf53* and cis-eQTL-associated genes were analyzed using the GeneMANIA tool [34].
- The atSNP Function Prediction online tool (http://atsnp.biostat.wisc.edu/search (accessed on 15 May 2024)) was utilized to assess how *C19orf53* SNPs affect the gene’s affinity for transcription factors (TFs) based on the presence of reference or alternative alleles [35]. This tool evaluated the impact of SNPs on TF-DNA interactions using a positional weight matrix to calculate the degree of influence.
- The Gene Ontology online tool (http://geneontology.org/ (accessed on 15 May 2024)) allowed us to analyze the involvement of transcription factors associated with the reference and SNP alleles in overrepresented biological processes related to IS pathogenesis [36]. This tool helped identify biological functions regulated by TFs linked to *C19orf53* SNPs.
- The Cerebrovascular Disease Knowledge Portal (CDKP) (https://cd.hugeamp.org/ (accessed on 16 May 2024)) and Cardiovascular Disease Knowledge Portal (https://cvd.hugeamp.org/ (accessed on 16 May 2024)) integrate and analyze genetic association data from major consortia to study cardio- and cerebrovascular diseases, aiding in the exploration of associations between *C19orf53* SNPs and atherosclerosis-related conditions and risk factors for IS (like low density lipids, body mass index, total cholesterol) [37].

## 3. Results

The *C19orf53* gene is expressed in various parts of the brain as well as in blood vessels and whole blood. *C19orf53* gene expression levels (MeTPM) range from 68.48 to 140.8 in brain tissues, range from 120.5 to 136.0 in blood vessels, and are 45.11 in whole blood (Supplementary Figure S2).

### 3.1. C19orf53 SNPs and the Ischemic Stroke Risk: An Analysis of Associations

The genotype frequencies of rs10104, rs11666524, rs346157, rs346158, rs2901077, rs2277947, and rs8107914 *C19orf53* in study groups are presented in Table S2. In the control group, the genotype frequencies of all studied SNPs corresponded to the Hardy–Weinberg equilibrium (*p* > 0.05).

The analysis of the total sample revealed associations between SNPs and the risk of an IS (Table S2): rs346157 (EA G; OR_(R)_ = 1.30; 95% CI_(R)_ = 1.03–1.64; P_(R)_ = 0.03), rs346158 (EA C; OR_(R)_ = 1.46; 95% CI_(R)_ = 1.01–2.11; P_(R)_ = 0.04), and rs2901077 (EA T; OR_(R)_ = 2.49; 95% CI_(R)_ = 1.11–5.57; P_(R)_ = 0.02). However, these associations were weak and did not remain significant after applying Bonferroni correction for the number of regression models (three) (Table S3).

We analyzed the subgroups depending on smoking status, obesity, fresh fruit and vegetable intake, and physical activity level (Table S4). Our data reveal associations between specific genetic variants and the risk of an IS under the three following different conditions: obesity, low physical activity, and low fruit and vegetable intake (Table 2).

First, in obese patients (BMI ≥ 30), the risk of an IS increases four SNPs *C19orf53*, with P being significant in both recessive (R) and log-additive models (A): rs10104 (EA G; OR_(R)_ = 4.7; 95% CI_(R)_ = 2.25–9.82; P_R_ = 0.0003 (P_(R) bonf_ = 0.0009) and OR_(A)_ = 1.82; 95% CI_(A)_ = 1.22–2.72; P_(A)_ = 0.004 (P_(A) bonf_ = 0.01)), rs11666524 (EA A; OR_(R)_ = 3.88; 95% CI_(R)_ = 1.88–8.02; P_(R)_ = 0.001 (P_(R) bonf_ = 0.003) and OR_(A)_ = 1.76; 95% CI_(A)_ = 1.19–2.6; P_(A)_ = 0.006 (P_(A) bonf_ = 0.02)), rs346158 (EA C; OR_(R)_ = 3.73; 95% CI_(R)_ = 1.81–7.69; P_(R)_ = 0.002 (P_(R) bonf_ = 0.006) and OR_(A)_ = 1.65; 95% CI_(A)_ = 1.11–2.44; P_(A)_ = 0.015 (P_(A) bonf_ = 0.045)), and rs2277947 (EA A; OR_(R)_ = 4.11; 95% CI_(R)_ = 1.98–8.53; P_(R)_ = 0.0008 (P_(R) bonf_ = 0.002) and OR_(A)_ = 1.82; 95% CI_(A)_ = 1.22–2.7; P_(A)_ = 0.004 (P_(A) bonf_ = 0.01)) (Table 2).

Second, in subjects with low physical activity, the following genetic variants were discovered to be related to the increased risk of an IS: rs10104 (EA G; OR_(R)_ = 1.91; 95% CI_(R)_ = 1.19–3.07; P_(R)_ = 0.009 (P_(R) bonf_ = 0.03)) and rs11666524 (EA A; OR_(R)_ = 1.8; 95% CI_(R)_ = 1.13–2.84; P_(R)_ = 0.016 (P_(R) bonf_ = 0.048)) (Table 2).

Third, under the condition of low fruit and vegetable consumption, the following genetic variants were associated with an increased risk of an IS: rs11666524 (EA A; OR_(R)_ = 1.81; 95% CI_(R)_ = 1.2–2.75; P_(R)_ = 0.006 (P_(R) bonf_ = 0.02)), rs346157 (EA G; OR_(R)_ = 1.42; 95% CI_(R)_ = 1.08–1.86; P_(R)_ = 0.012 (P_(R) bonf_ = 0.036)), rs346158 (EA C; OR_(R)_ = 1.93; 95% CI_(R)_ = 1.29–2.89; P_(R)_ = 0.0017 (P_(R) bonf_ = 0.005)), and rs2277947 (EA A; OR_(R)_ = 1.81; 95% CI_(R)_ = 1.18–2.76; P_(R)_ = 0.0074 (P_(R) bonf_ = 0.02)) (Table 2).

### 3.2. C19orf53 SNPs and Clinical Parameters

**Figure 2:**
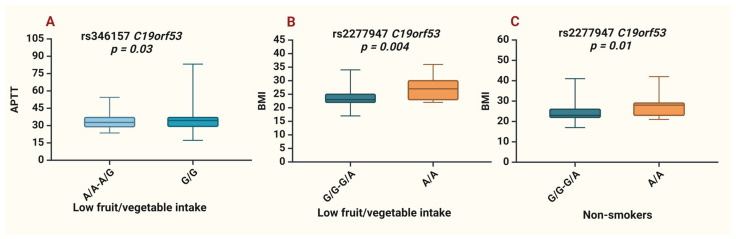
Associations of *C19orf53* SNPs and clinical parameter. (**A**)—APTT values for rs346157 in patients with low fruit and vegetable consumption; (**B**)—BMI data for rs2277947 in patients with low fruit/vegetable intake; (**C**)—BMI values for rs2277947 in non-smoking patients.

Upon analyzing the clinical characteristics in patients with IS, it was observed that allele G rs346157 was associated with lower APTT in patients with low fruit/vegetable intake (*p* = 0.03); allele A rs2277947 was associated with a higher BMI in patients with low fruit/vegetable intake (*p* = 0.004) and in non-smokers (*p* = 0.01) (Figure 2, Table S5).

### 3.3. The Role of Bioinformatic Tools in Substantiating the Results

For the functional annotation of SNPs and the bioinformatic analysis of the role of the *C19orf53* gene in the pathogenetic mechanisms of ischemic stroke, we used a range of tools aimed at analyzing the relationship of SNPs with transcription factors, expression quantitative trait loci, and histone modifications. Additionally, gene–gene and protein–protein interaction networks were constructed.

#### 3.3.1. QTL-Effects

Based on data from the eQTLGene browser SNPs, linked to a higher risk of IS, specifically rs10104 (effect allele G), rs11666524 (effect allele A), rs346158 (effect allele C), and rs2277947 (effect allele A) are associated with reduced levels of expression of both *C19orf53* and *MRI1* as well as increased levels of *CCDC130* and *ZSWIM4* expression in peripheral blood (Table 3). On the other hand, rs346157 (effect allele G) is also linked to reduced expression levels of *C19orf53* and *MRI1* and an increase in *CCDC130* expression but does not affect the expression levels of *ZSWIM4* (Table 3).

GTEx Portal data reveal correlations between IS-associated SNPs and the reduced expression levels of *C19orf53* in brain and adipose tissues as well as in arteries through eQTL effects. Additionally, the expression of the *MRI1* gene shows a decrease in arteries, including the aorta and tibial arteries, as well as in the brain cortex and subcutaneous adipose tissue. These changes in *MRI1* expression are associated with the presence of rs10104, rs11666524, rs346158, and rs2277947 SNPs (Table 4).

**Figure 3:**
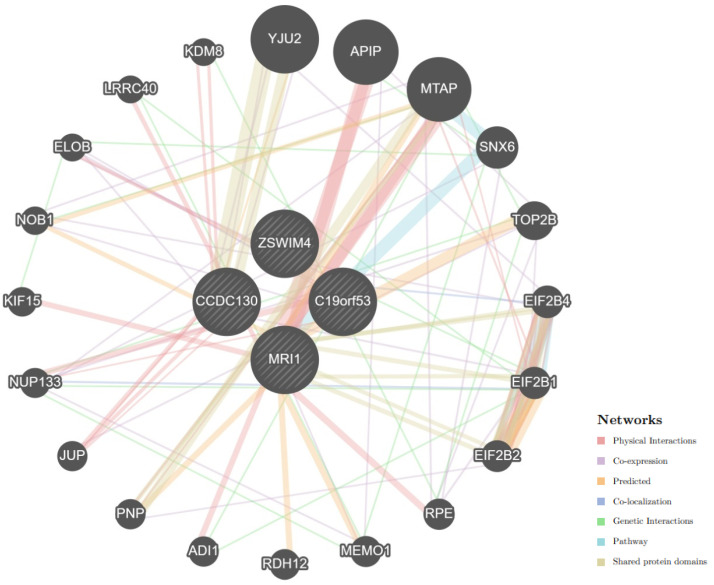
Interactions between *C19orf53* and cis-eQTL-associated genes (24 total genes, 4 searched genes, 20 related genes, 125 total links). Note: Genes searched are indicated with stripes.

Using the GeneMania resource, an analysis of interactions between *C19orf53* and cis-eQTL-associated genes was carried out (*MRI1*, *CCDC130*, *ZSWIM4*). It turned out that they interact through 20 related genes and 125 total links, mainly through physical interactions (77,64%), co-expression (8,01%), prediction (5.37%), co-localization (3.63%), genetic interactions (2.87%), pathways (1.88%), and shared protein domains (0.60%) (Figure 3, Table S6).

The analysis of the joint functions of genes in a network of interactions of *C19orf53* and cis-eQTL-related genes showed their participation in such processes significant for the development of an IS as oligodendrocyte differentiation, sulfur amino acid/aspartate family amino acid metabolic process (Table S7).

#### 3.3.2. Histone Modifications

Utilizing the bioinformatics resource HaploReg (v4.2), we conducted an analysis of histone modifications associated with SNPs identified in our study as being linked to an increased risk of IS.

Specifically, all SNPs linked to IS are located in the region of DNA binding to histone H3, characterized by mono-methylation at the 4th lysine residue of the histone H3 protein (H3K4me1) in both blood cells and brain tissues. Furthermore, SNPs rs10104, rs11666524, and rs2901077 are located in the region of DNA binding to H3K4me3 in both brain regions and peripheral blood.

These histone modifications are further complemented by the effects of H3K27ac, marking enhancers, and the acetylation at the 9th lysine residues of the H3K9ac, marking promoters in both blood cells and brain tissues for the SNPs rs10104, rs11666524, rs346157, rs346158, and rs2901077.

As for SNP rs2277947, it is localized within DNA region that binds to both H3K4me1 and H3K4me3 but exclusively in blood cells. The impact of these histone tags is further enhanced by the presence of H3K27ac, marking enhancer and H3K9ac, marking promoters specifically in blood cells.

Notably, SNP rs10104 is additionally found within DNA regions that are hypersensitive to DNase-1 in blood cells (Table 5). The significant effect of rs10104 *C19orf53* on DNA binding to regulatory proteins EGR1, POL24H8, TAF1, POL2, CFOS, ETS1, MAX, POL2B, ZBTB7A, and ZNF263 (https://pubs.broadinstitute.org/mammals/haploreg/detail_v4.2.php?query=&id=rs10104) (accessed on 14 May 2024) should also be noted.

#### 3.3.3. Analysis of Transcription Factors

The risk allele G of rs10104 *C19orf53* is associated with the generation of DNA-binding sites for 79 transcription factors (TFs) (Table S8). These TFs are involved in five overrepresented biological processes: the interleukin-9-mediated signaling pathway (GO:0038113; FDR = 0.0245), neuron fate determination (GO:0048664; FDR = 0.0375), the positive regulation of neuron differentiation (GO:0045666; FDR = 0.0105), the regulation of endothelial cell proliferation (GO:0001936; FDR = 0.0353), and the negative regulation of neurogenesis (GO:0050768; FDR = 0.0424) (Table S8).

Nineteen TFs, binding to the risk allele A of rs11666524 *C19orf53*, are concurrently implicated in two overrepresented GO: the positive regulation of neuron differentiation (GO:0045666; FDR = 0.00146) and astrocyte fate commitment (GO:0060018; FDR = 0.00306) (Table S9).

The risk allele G of rs346157 *C19orf53* produces DNA-binding regions for 22 transcription factors, collectively engaged in four overrepresented GOs related to cell proliferation, oxidative stress, cell signaling, and apoptosis: positive regulation of glial cell proliferation (GO:0060252; FDR = 0.0259), cellular response to reactive oxygen species (GO:0034614; FDR = 0.0248), response to hypoxia (GO:0001666; FDR = 0.0197), and mononuclear cell differentiation (GO:1903131; FDR = 0.0371) (Table S10). On the other hand, the protective allele A of rs346157 *C19orf53* provides DNA-binding sites for 63 TFs, which are together participate in 11 GOs associated with the blood vessel morphogenesis (GO:0048514; FDR = 0.00756), regulation of the vascular tone (cellular response to angiotensin (GO:1904385; FDR = 0.00475), neurogenesis (central nervous system development (GO:0007417; FDR = 0.0179)), hypoxia (cellular response to hypoxia (GO:0071456; FDR = 0.00167)), oxidative stress (cellular response to oxidative stress (GO:0034599; FDR = 0.0154)), response to heat (regulation of cellular response to heat (GO:1900034; FDR = 0.0492)), cell signaling (signal transduction by p53 class mediator (GO:0072331; FDR = 0.0071), positive regulation of protein binding (GO:0032092; FDR = 0.00685)), proteostasis (PERK-mediated unfolded protein response (GO:0036499; FDR = 0.015)), and apoptosis (positive regulation of neuron apoptotic process (GO:0043525; FDR = 0.00157), intrinsic apoptotic signaling pathway (GO:0097193; FDR = 0.0462)) (Table S10).

Lastly, the risk allele A of rs2277947 *C19orf53* creates DNA-binding sites for 32 TFs, associated with overrepresented GO such as glial cell fate commitment (GO:0021781; FDR = 0.0235), the positive regulation of neuron differentiation (GO:0045666; FDR = 0.0341), and blood vessel development (GO:0001568; FDR = 0.0107) (Table S11).

#### 3.3.4. Bioinformatic Analysis of the Associations of *C19orf53* SNPs with IS-Related Phenotypes

According to the bioinformatic tools CDKP and CVDKP, the IS-associated *C19orf53* SNPs are linked with ischemic stroke risk and the severity of IS as well as with obesity and a number of laboratory parameters, characterizing the lipid metabolism (Table 6). Particularly noteworthy is the identification of a connection between these SNPs and arterial fibrillation along with indicators of dyslipidemia (triglyceride-to-HDL ratio, serum ApoB),—significant risk factors for IS.

#### 3.3.5. Protein–Protein Interactions

Nine proteins having the most prominent interactions with *C19orf53* were found after searching for the primary functional partners of *C19orf53* using the STRING database data (proteins of the first shell of interactors): POLR2J, C19orf67, CCDC124, COPS9, NDUFA11, NDUFB7, SERF2, RNF181, C9orf16 (PPI enrichment *p*-value = 0.00201) (Supplementary Figure S3, Table S13).

Together, *C19orf53* and its main functional partners are involved in six biological processes, reflecting predominantly ATP synthesis (for example: «mitochondrial respiratory chain complex I assembly» (GO:0032981, FDR = 0.0032), «mitochondrial electron transport, NADH to ubiquinone» (GO:0006120, FDR = 0.0121); «proton motive force-driven mitochondrial ATP synthesis» (GO:0042776, FDR = 0.0032)); proteostasis («proteasome-mediated ubiquitin-dependent protein catabolic process» (GO:0043161; FDR = 0.00083)); and metabolic pathways («nucleobase-containing compound biosynthetic process» (GO:0034654; FDR = 0.0038), «nitrogen compound metabolic process» (GO:0006807; FDR = 0.0354)) (Table S14).

## 4. Discussion

**Figure 4:**
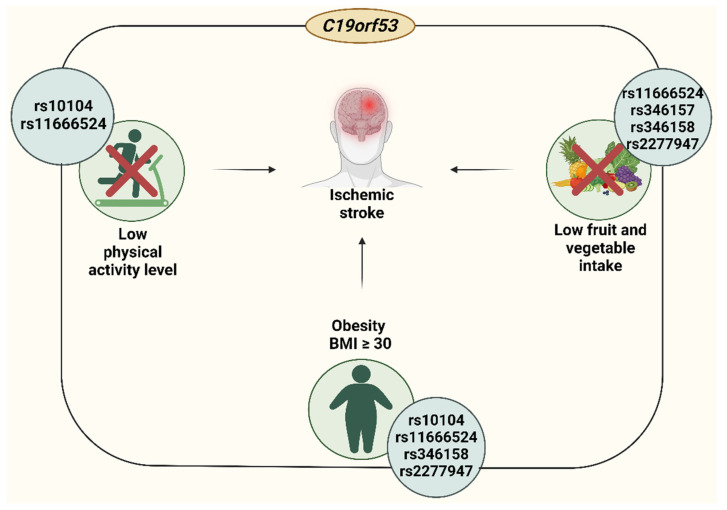
The interplay of polymorphic variants of the *C19orf53* gene and environmental factors in the risk of IS development.

Here, providing genetic evidence that SNPs rs10104, rs11666524, rs346157, rs346158, and rs2277947 *C19orf53* are associated with IS risk, we report that *C19orf53* is involved in the pathogenesis of stroke. In particular, BMI, physical activity, and the consumption of fruit and vegetables act as modifiers of the associations of SNPs rs10104, rs11666524, rs346157, rs346158, and rs2277947 (Figure 4).

Moreover, genotypes A/A-A/G vs. G/G for rs346157 exhibited longer activated partial thromboplastin time (APTT) in patients with insufficient fruit and vegetable intake, and genotypes G/G-G/A vs. A/A for rs2277947 were associated with a higher BMI in non-smokers and patients with low fruit and vegetable intake. Interestingly, another Hero protein member, SERBP1, has also been associated with shorter APTT and a higher BMI [22]. A reduction in APTT, which measures the time required for fibrin formation, is associated with an elevated risk of ischemic stroke, greater disease severity, and poorer outcomes [38]. Thus, rs346157 may be linked to excessive thrombosis.

SNP rs10104 A/G is a missense variant leading to the substitution of Lys39Arg at codon 39 of the C19orf53 protein. It is noteworthy that rs10104 is an MRI1 Downstream Variant, significantly affecting the expression of *C19orf53* (Table 4 and Table 5). SNPs rs11666524, rs346157, and rs346158 localized in introns, and rs2277947 is a 3′prime UTR variant.

Due to the fact that the use of PubMed and Google Scholar search resources did not allow us to detect studies aimed at investigating the associations of the *C19orf53* SNPs with the development of IS/cardiovascular diseases, we used the bioinformatics analysis to interpret the effects of these genetic variants. Several compelling factors underscore IS-associated SNPs significance in unraveling the mechanisms behind IS.

### 4.1. C19orf53 SNPs and the IS Risk: Underlying Mechanisms

**Figure 5:**
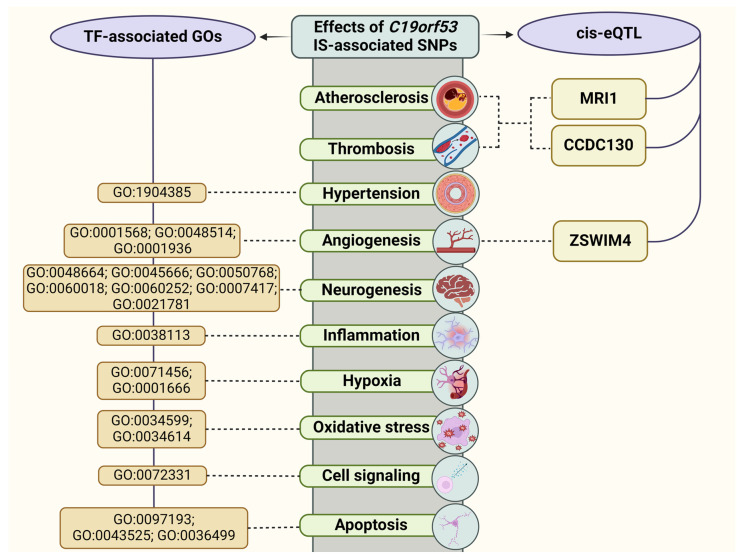
The effects of *C19orf53* SNPs associated with IS.

Based on cis-eQTL effects and the analysis of transcription factors of *C19orf53* SNPs, associated with IS risk, we distinguished the main mechanisms contributing to disease development (Figure 5).

The IS-associated *C19orf53* SNPs exhibit a high level of regulatory potential, exerting profound effects on histone modifications within brain and blood tissues. Notably, they prominently influence histone H3 by inducing trimethylation at the 4th lysine residue, and they also impact the H3K27ac.

Furthermore, it is worth highlighting that the risk alleles of the identified SNPs, which were found to correlate significantly with IS in our study, exhibit associations beyond IS alone, according to the Cerebrovascular Disease Knowledge Portal. Specifically, these SNPs are associated with an increased risk of all ischemic strokes, as indicated by rs10104, rs11666524, rs346157, rs346158, and rs2277947. Moreover, rs10104, rs11666524, rs346158, and rs2277947 also play a role in determining the severity of stroke, as evidenced by their correlation with modified Rankin scale scores. Additionally, SNPs rs10104, rs11666524, rs346158, and rs2277947 are implicated in atrial fibrillation or flatter, further underscoring their relevance in IS pathogenesis [39]. Complementary, data collected from the Cardiovascular Disease Knowledge Portal reveal that protective alleles of *C19orf53* SNPs exhibit a distinct influence on lipid metabolism, increasing triglyceride to HDL ratio and lowering serum ApoB levels, all of which are critical factors in atherosclerosis and IS development [40].

In ischemic conditions, established molecular mechanisms and cellular processes, including excitotoxicity, oxidative and nitrative stress, inflammation, and apoptosis, contribute to cell death [41]. Recognizing the potential involvement of *C19orf53* polymorphisms in these pathophysiological processes becomes crucial. The IS-associated SNPs of *C19orf53*, which display complex interactions with TFs, are providing insights into their involvement in these important cellular processes. Notably, the risk allele G of rs10104 *C19orf53* generates binding sites for TFs involved in inflammation, neurogenesis, and the regulation of endothelial cell proliferation. Similarly, the risk allele A of rs11666524 *C19orf53* creates binding sites for TFs primarily associated with neuron differentiation and astrocyte fate commitment. Moreover, the risk allele G of rs346157 *C19orf53* provides binding sites for TFs with roles in glial cell proliferation, responses to hypoxia and oxidative stress response, cell signaling, and apoptosis, while its protective A allele introduces binding sites for TFs associated with vasculo- and neurogenesis, the regulation of the vascular tone, hypoxia and oxidative stress response, heat response, cell signaling, proteostasis, and apoptosis. It is worth noting that TFs binding to the DNA site created by protective allele A rs346157 participate in the PERK-mediated pathway. This pathway is involved in protecting the brain from an ischemia–reperfusion injury by inhibiting ER stress-induced apoptosis [42]. Additionally, the risk allele A of rs2277947 *C19orf53* is linked to TFs associated with vasculo- and neurogenesis. In summary, these allele-specific binding sites highlight *C19orf53*’s involvement in pivotal biological processes related to neuron development and differentiation, vasculogenesis, the regulation of vascular tone, and oxidative stress response as well as response to hypoxia, response to heat, proteostasis, inflammation, and the regulation of apoptosis pathways, playing a pivotal role in the molecular pathogenesis of IS [43,44]. Consequently, we can hypothesize that *C19orf53* not only increases the risk of an IS, as established by our genetic analysis, but is also involved in the complex mechanisms governing the survival of neurons, glia, and endothelial cells by means of gains and losses of binding sites for TFs.

To further expand on the involvement of C19orf53 within cellular functions as well as pathophysiological processes, we analyzed its protein–protein interaction network, shedding light on its principal functional partners and their critical roles. First, C19orf53 and its principal functional partners participate in processes related to cellular energy production, such as “mitochondrial respiratory chain complex I assembly”, “mitochondrial electron transport, NADH to ubiquinone”, and “proton motive force-driven mitochondrial ATP synthesis”, emphasizing their significance in maintaining energy balance and mitochondrial functionality and potentially in excitotoxicity [45]. Second, their association with “proteasome-mediated ubiquitin-dependent protein catabolic process” signifies a role in maintaining protein homeostasis by aiding in the degradation of damaged proteins [46], a pathway triggered by ischemia [47]. Furthermore, proteins’ involvement in “nucleobase-containing compound biosynthetic process” underscores their contributions to nucleotide and nucleic acid metabolism, crucial for DNA and RNA synthesis, ensuring proper cellular function. It is worth noting that C19orf53 interacts with C9orf16 and SERF2, both correlated with IS risk in our previous studies [20,21]. Collectively, these insights indicate that C19orf53 and its functional partners are involved in pathophysiological cellular processes, thereby contributing to the underlying mechanisms of IS.

Examining cis-eQTL effects of the IS-associated SNPs, a common theme emerges where these genetic variants (rs10104, rs11666524, rs346157, rs346158, and rs2277947) together contribute to the downregulation of *C19orf53* expression in various tissue types, including blood, brain, and adipose tissues and arteries. Moreover, these SNPs, with the exception of rs346157, exhibit a similar pattern of lowering the expression of the *MRI1* gene in arteries, adipose tissues, and the brain cortex. MRI1 plays a vital role as an enzyme involved in the methionine de novo and salvage pathway as well as the metabolism of sulfur amino acid (https://www.genecards.org, assessed 25 May 2024). We hypothesize that the reduction in MRI1 production resulting from these SNP-driven alterations may have profound consequences, disrupting these essential metabolic pathways. Notably, the methionine cycle, in which MRI1 plays a role, holds significant importance in stroke and cardiovascular diseases through the regulation of homocysteine levels [48]. Additionally, sulfur-containing amino acids and their derivatives can regulate body weight, adipogenesis, lipolysis, and glucose metabolism [49], providing a link to associations between *C19orf53* SNPs and an increased BMI.

In contrast, *ZSWIM4* displays an increased expression in blood affected by the risk alleles of rs10104, rs11666524, rs346158, and rs2277947. Expanding on the role of ZSWIM4 in the context of IS, it is important to highlight its involvement in the ubiquitination pathway, as demonstrated in studies [50]. Mechanically, ZSWIM4 has been found to have physical interactions with Smad1, leading to the ubiquitination and subsequent degradation of Smad1 [51]. This interaction holds significance within the broader context of IS, as Smad1 intersects with the regulation of angiogenesis following ischemia/reperfusion. Specifically, the TGF-β1/ALK1/Smad1/5/8 signaling pathway has been identified as a key player in mediating angiogenic processes in response to ischemic events [52,53]. By influencing the ubiquitination and degradation of Smad1, the increased expression of *ZSWIM4,* caused by the effects of *C19orf53* SNPs, in blood may contribute to the downregulation of this critical pathway, potentially impacting the post-ischemic angiogenic response. Conversely, the presence of IS-associated alleles of *C19orf53* is associated with higher expression levels of *CCDC130* in blood. This observation is of particular significance since members of the CCDC protein family, to which *CCDC130* belongs, have been demonstrated to have roles in lipid metabolism and inflammation [54], atherosclerosis, and thrombus formation [55,56]. This intriguing association suggests a potential link between *C19orf53* and the development of IS through the modulation of *CCDC130* expression and its implications in pathological pathways.

The analysis of the joint functions of genes in a network of interactions of *C19orf53* and cis-eQTL-related genes showed that their participation in such processes was significant for the development of IS as oligodendrocyte differentiation, a sulfur amino acid metabolic process. Researchers have already shown the significant role of sulfur amino acids in atherosclerosis [57], and it was found that sulfur-containing amino acid metabolites are associated with the risk of ischemic stroke and fibrin clot properties [58,59] and affect the concentration of glutathione [60], a powerful antioxidant, the level of which can largely determine the risk of an IS [61]. Moreover, plasma sulfur amino acids are obesogenic [62].

Additionally, to further emphasize *C19orf53*’s potential impact on the risk of stroke, there exists a significant relationship between *C19orf53* and Heat Shock Factor 1 (HSF1) discovered by the authors [16]. Notably, experiments involving *HSF1* knockout demonstrated a substantial reduction in *C19orf53* expression, underscoring the regulatory influence of HSF1 on *C19orf53*. HSF1, known as a heat shock factor, plays a pivotal role in orchestrating stress-induced transcription and governing diverse physiological processes within cells. Its central position in maintaining cellular homeostasis is well-established, primarily through its ability to transactivate genes responsible for encoding heat shock proteins (HSPs). This newfound association underscores the implication of C19orf53 in the heat stress response, a pathway of paramount importance in the context of ischemia/reperfusion events and stroke pathogenesis.

### 4.2. Insuficient Fruit and Vegetable Intake-Linked Associations of C19orf53

The correlation of SNPs rs10104, rs11666524, rs346157, rs346158, and rs2277947 with IS was significantly modified by the levels of fresh vegetable and fruit consumption. The link between a reduced intake of fresh vegetables and fruits and increased oxidative stress has been well established. Oxidative stress has been extensively studied and demonstrated to play a significant role in the risk of IS and related phenotypes in numerous investigations. Plant-rich diets, typically rich in phenolic acids, flavonoids, and carotenoids, are known for their potent antioxidant properties. These antioxidants effectively combat excessive reactive oxygen species in the body, safeguarding cells from their negative effect and consequently lowering the risk of cardiovascular diseases.

The analysis of transcriptomic profiling data has unveiled a significant correlation between *C19orf53* and key genes involved in oxidative stress response, shedding light on its potential role in the risk of stroke. Notably, *C19orf53* exhibits strong correlations with *PARK7*, *SOD1*, and *ROCK1*, all playing a crucial part in autophagic proteolysis and autophagosome formation [63,64,65]. PARK7, in particular, is reported as a multifaceted player in the cellular defense against oxidative stress. Acting as a redox-sensitive chaperone and an oxidative stress sensor, PARK7 is instrumental in safeguarding neurons against oxidative stress-induced cells. Moreover, it prevents the formation of advanced glycation end-products, eliminates hydrogen peroxide, and maintains proper mitochondrial functions, including the autophagy of dysfunctional mitochondria. Additionally, PARK7 regulates inflammatory responses in astrocytes and neuronal cells. *SOD1*, another gene strongly correlated with *C19orf53*, plays a key role in antioxidant defense [66]. Its protein product is a scavenger of harmful superoxide radicals by converting them into molecular oxygen and hydrogen peroxide. Lastly, *ROCK1*, while not directly involved in oxidative stress response, acts as a negative regulator of VEGF-induced angiogenic endothelial cell activation, which can have implications in vascular health and stroke risk. This intricate web of correlations underscores the potential involvement of *C19orf53* in oxidative stress pathways and its connection to the risk of stroke.

### 4.3. Low Physical Activity-Related Correlates of C19orf53

Numerous studies have consistently demonstrated the benefits of physical exercise, establishing a positive connection to better heart and vessel health [67]. Exercising increases the expression levels of HSPs [68] as well as HSF1 [69]. As previously mentioned, *HSF1* regulates the expression of *C19orf53*. The upregulation of *C19orf53* levels can yield positive effects, as it participates in stress responses, leading to antioxidant [70] and anti-inflammatory effects [71,72] in brain tissues. Another protein linked to C19orf53, as discussed earlier, is VEGF. The expression of *VEGF* increases in response to exercise [73,74,75,76,77]. VEGF is significant for neurovascular integrity, participating in endothelial cell proliferation and angiogenesis and in securing neurotrophic, neuroprotective, and neurogenic effects [78]. The interplay of exercise-induced molecular responses involving HSPs, HSF1, C19orf53, and VEGF results in an increased risk of an IS for patients with low physical activity levels carrying rs10104 and rs1166524 *C19orf53* SNPs.

### 4.4. Obesity-Related Correlates of C19orf53

Obesity had a substantial impact on the relationship between rs11666524, rs346158, and rs2277947 and the risk of an IS. Patients with a BMI of 30 or greater exhibited significant associations. The Bonferroni adjustment leveled associations in the subgroup with BMI < 30, except for rs10104 *C19orf53*, which maintained a weak connection at *p* = 0.046. First, overweight and obesity have been associated with a gradually greater risk of stroke, as revealed by a vast amount of research [79,80,81]. A bioinformatics study of PheWAS, carried out with the support of Knowledge Portals resources, indicated the influence of all IS-related SNPs on the abdominal fat ratio; rs346157 also exhibited a substantial risk effect in the phenotype “obese vs. controls” (Table 6). Furthermore, our research shows that these SNPs have significant cis-eQTL-mediated effects on both *C19orf53* and *MRI1* expression levels. MRI1 has been found to alter the metabolism of sulfur-containing amino acids and their derivatives, which play important roles in body weight regulation, adipogenesis, lipolysis, and glucose metabolism [49], implying a relationship between the *C19orf53* SNPs and an increased BMI. Additionally, the assessment of SNPs’ histone modifications shows that obesity-related SNPs are characterized by high levels of epigenetic modulation in adipose tissue. Further studies of *C19orf53* SNPs are required, primarily to analyze their associations with the risk of obesity and BMI, which will allow us to determine whether IS-related *C19orf53* SNPs are characterized by pronounced horizontal pleiotropic effects on the development of both IS and obesity or whether their effect on IS is due to mediators’ effects, reflecting their influence on the risk of IS through excess body weight.

## 5. Conclusions

The present study provides genetic evidence of associations between *C19orf53* polymorphisms and IS in the Caucasian population of central Russia. The results were supported with a bioinformatic assessment, revealing genome-, epigenome-, and proteome-wide effects of *C19orf53* polymorphisms as well as an established link between SNPs in the *C19orf53* gene and cerebrovascular disease-related phenotypes.

## 6. Limitations of the Study

Our study has several limitations. First, we did not study the level of *C19orf53* expression, which does not allow us to establish the effect of the studied SNPs on the level of *C19orf53* mRNA. Second, we were limited in our analysis of environmental risk factor scores, especially in the control group, which did not allow us to conduct a model-based multifactor dimensionality reduction (MDR) analysis of gene–environment interactions. Third, we did not replicate our data in another independent population sample.

## Figures and Tables

**Table 1:** General clinical characteristics of IS patients and controls.

Baseline and Clinical Characteristics		IS Patients (n = 947)	Controls (n = 1308)	*p*-Value
Age, Me [Q1; Q3]		63 [55; 70]	59 [53; 66]	**<0.001**
Gender	Males, N (%)	524 (55.33%)	614 (46.9%)	**<0.001**
	Females, N (%)	423 (44.67%)	694 (53.1%)	
Smoking	Yes, N (%)	469 (49.52%)	413 (31.6%)	**<0.001**
	No, N (%)	478 (50.48%)	895 (68.4%)	
Low physical activity	Yes, N (%)	354 (39.91%)	ND	
	No, N (%)	533 (60.09%)		
Low fruit/vegetable consumption	Yes, N (%)	416 (46.9%)	ND	
	No, N (%)	471 (53.1%)		
Coronary artery disease	Yes, N (%)	116 (13.08%)	-	
	No, N (%)	771 (86.92%)	-	
Obesity	Yes, N (%)	61 (6.4%)	ND	
	No, N (%)	530 (56%)		
	ND, N (%)	356 (37.6%)		
Family history of cerebrovascular diseases	Yes, N (%)	312 (35.29%)	ND	
	No, N (%)	572 (64.71%)	ND	
Age at onset of stroke, Me [Q1; Q3]		61 [54; 70] (n = 904)	-	
Number of strokes including event in question	1, N (%)	800 (88.59%)	-	
	2, N (%)	89 (9.86%)	-	
	3, N (%)	14 (1.55%)	-	
Stroke localization	Right/left middle cerebral artery basin, N (%)	750 (83.24%)	-	
	Vertebrobasilar basin, N (%)	151 (16.76%)	-	
Area of lesion in stroke, mm^2^, Me [Q1; Q3]		105 [30; 468] (n = 883)	-	
Total cholesterol, mmol/L, Me [Q1; Q3]		5.2 [4.4; 5.9] (n = 608)	ND	
Triglycerides, mmol/L, Me [Q1; Q3]		1.3 [1.1; 1.8] (n = 601)	ND	
Glucose level, mmol/L, Me [Q1; Q3]		4.8 [4.3; 5.5] (n = 892)	ND	
Prothrombin time, seconds, Me [Q1; Q3]		10.79 [10.14; 11.70] (n = 880)	ND	
International normalized ratio, Me [Q1; Q3]		1 [0.94; 1.09] (n = 596)	ND	
Activated partial thromboplastin time, seconds, Me [Q1; Q3]		32.7 [29; 37] (n = 599)	ND	

Statistically significant differences between groups are indicated in bold; ND—no data.

**Table 2:** Statistically significant correlations between *C19orf53* SNPs and IS risk determined by BMI, fruit and vegetable intake, and physical activity.

SNP	N	Dominant Model		Recessive Model		Log-Additive Model		N	Dominant Model		Recessive Model		Log-Additive Model	
		OR (95% CI) ^1^	P (P_bonf_)	OR (95% CI) ^1^	P (P_bonf_)	OR (95% CI) ^1^	P (P_bonf_)		OR (95% CI) ^1^	P (P_bonf_)	OR (95% CI) ^1^	P (P_bonf_)	OR (95% CI) ^1^	P (P_bonf_)
	BMI ≥ 30							BMI < 30						
rs10104 *C19orf53* (A/G)	1286	1.52 [0.90–2.55]	0.12	**4.70** **[2.25–9.82]**	**0.0003** **(0.0009)**	**1.82** **[1.22–2.72]**	**0.004** **(0.01)**	1745	0.94 [0.76–1.16]	0.57 (1.0)	**1.60** **[1.02–2.51]**	**0.046** (1.0)	1.02 [0.86–1.22]	0.79 (1.0)
rs11666524 *C19orf53* (G/A)	1330	1.54 [0.92–2.58]	0.098	**3.88** **[1.88–8.02]**	**0.001** **(0.0** **0** **3)**	**1.76** **[1.19–2.60]**	**0.006** **(0.02)**	1792	0.94 [0.76–1.15]	0.55 (1.0)	**1.64** **[1.09–2.48]**	**0.02** (0.06)	1.04 [0.88–1.23]	0.68 (1.0)
rs346158 *C19orf53* (T/C)	1301	1.40 [0.84–2.35]	0.2	**3.73** **[1.81–7.69]**	**0.002** **(0.006)**	**1.65** **[1.11–2.44]**	**0.015** **(0.045)**	1769	0.90 [0.73–1.11]	0.32 (0.96)	**1.60** **[1.06–2.40]**	**0.03** (0.09)	1.01 [0.85–1.19]	0.94 (1.0)
rs2277947 *C19orf53* (G/A)	1242	1.60 [0.95–2.70]	0.079	**4.11** **[1.98–8.53]**	**0.0008** **(0.002)**	**1.82** **[1.22–2.70]**	**0.004** **(0.01)**	1699	0.98 [0.80–1.21]	0.87 (1.0)	**1.60** **[1.05–2.45]**	**0.03** (0.09)	1.06 [0.90–1.26]	0.48 (1.0)
	Low physical activity level (f+)							Normal physical activity level (f−)						
rs10104 *C19orf53* (A/G)	1597	0.86 [0.67–1.09]	0.21 (0.63)	**1.91** **[1.19–3.07]**	**0.009** **(0.0** **3** **)**	1.00 [0.82–1.22]	0.99 (2.3)	1774	0.93 [0.76–1.15]	0.5 (1.5)	1.14 [0.71–1.83]	0.6 (1.8)	0.97 [0.81–1.15]	0.7 (2.1)
rs11666524 *C19orf53* (G/A)	1623	0.85 [0.67–1.08]	0.18 (0.5)	**1.80** **[1.13–2.84]**	**0.016** **(0.048)**	0.99 [0.81–1.20]	0.91 (2.7)	1798	0.97 [0.79–1.20]	0.79 (2.4)	1.29 [0.83–2.00]	0.26 (0.8)	1.02 [0.86–1.21]	0.84 (2.5)
	Low fruit/vegetable intake (f+)							Normal fruit/vegetable intake (f−)						
rs11666524 *C19orf53* (G/A)	1739	0.97 [0.78–1.21]	0.81 (2.4)	**1.81** **[1.20–2.75]**	**0.006** **(0.0** **2** **)**	1.08 [0.91–1.29]	0.37 (1.1)	1682	0.86 [0.69–1.09]	0.21 (0.6)	1.13 [0.68–1.86]	0.64 (1.9)	0.92 [0.76–1.11]	0.38 (1.1)
rs346157 *C19orf53* (A/G)	1752	0.91 [0.73–1.13]	0.4 (1.2)	**1.42** **[1.08–1.86]**	**0.012** **(0.036)**	1.06 [0.91–1.24]	0.43 (1.3)	1699	1.04 [0.82–1.31]	0.76 (2.3)	1.14 [0.85–1.53]	0.4 (1.2)	1.06 [0.90–1.24]	0.51 (1.5)
rs346158 *C19orf53* (T/C)	1740	0.96 [0.77–1.19]	0.71 (2.1)	**1.93** **[1.29–2.89]**	**0.0017** **(0.005)**	1.09 [0.92–1.30]	0.33 (1.0)	1687	0.86 [0.68–1.08]	0.18 (0.5)	1.07 [0.65–1.76]	0.79 (2.4)	0.91 [0.75–1.10]	0.31 (0.9)
rs2277947 *C19orf53* (G/A)	1668	1.02 [0.82–1.27]	0.86 (2.6)	**1.81** **[1.18–2.76]**	**0.0074** **(0.02)**	1.12 [0.93–1.33]	0.23 (0.7)	1604	0.90 [0.71–1.14]	0.38 (1.1)	1.24 [0.75–2.04]	0.4 (1.2)	0.96 [0.79–1.17]	0.68 (2.0)

All calculations were based on the minor alleles (effect alleles); ^1^—odds ratio and 95% confidence interval; *p*-value; statistically significant differences are marked in bold. Effect alleles (EA) are highlighted.

**Table 3:** Relationship between *C19orf53* SNPs and cis-eQTL-mediated expression levels of genes in whole blood (according to browser eQTLGene) (https://www.eqtlgen.org/) (accessed on 14 May 2024).

SNP	Assessed Allele	Other Allele	Gene	*p*-Value	FDR	Z-Score	N Samples
rs10104 *C19orf53*							
rs10104	**G**	A	*C19orf53*	3.27 × 10^−310^	0	−46.0132	27007
rs10104	**G**	A	*MRI1*	3.27 × 10^−310^	0	−38.6135	26793
rs10104	**G**	A	*CCDC130*	1.10 × 10^−19^	0	9.0787	27007
rs10104	**G**	A	*ZSWIM4*	8.02 × 10^−11^	0	6.5002	21718
rs11666524 *C19orf53*							
rs11666524	**A**	G	*C19orf53*	3.27 × 10^−310^	0	−46.2734	27279
rs11666524	**A**	G	*MRI1*	3.27 × 10^−310^	0	−39.214	27065
rs11666524	**A**	G	*CCDC130*	6.37 × 10^−20^	0	9.1379	27279
rs11666524	**A**	G	*ZSWIM4*	5.99 × 10^−11^	0	6.5441	21990
rs346157 *C19orf53*							
rs346157	**G**	A	*C19orf53*	2.14 × 10^−214^	0	−31.2513	27274
rs346157	**G**	A	*MRI1*	9.14 × 10^−150^	0	−26.0647	27060
rs346157	**G**	A	*CCDC130*	2.22 × 10^−36^	0	12.5961	27274
rs346158 *C19orf53*							
rs346158	**C**	T	*C19orf53*	3.27 × 10^−310^	0	−44.6143	26890
rs346158	**C**	T	*MRI1*	3.27 × 10^−310^	0	−38.1044	26676
rs346158	**C**	T	*CCDC130*	9.10 × 10^−18^	0	8.5849	26890
rs346158	**C**	T	*ZSWIM4*	3.91 × 10^−11^	0	6.6077	21601
rs2277947 *C19orf53*							
rs2277947	**A**	G	*C19orf53*	3.27 × 10^−310^	0	−46.393	27395
rs2277947	**A**	G	*MRI1*	3.27 × 10^−310^	0	−39.1083	27181
rs2277947	**A**	G	*CCDC130*	5.30 × 10^−20^	0	9.1579	27395
rs2277947	**A**	G	*ZSWIM4*	3.56 × 10^−11^	0	6.6213	22106

Effect allele are marked in bold.

**Table 4:** The impact of SNPs in genes encoding *C19orf53*, gene expression (cis-eQTL) in blood vessels, and brain and blood cells (according to the GTEx Portal browser, https://gtexportal.org) (accessed on 14 May 2024).

SNP	GTEx Portal Data (https://gtexportal.org)			
	Gene Expressed	*p*-Value	Effect (NES)	Tissue
rs10104 *C19orf53* (A/**G**)	*C19orf53*	**2.7 × 10** **^−18^**	↓ (−0.23)	Artery—Aorta
	*MRI1*	**3.0 × 10** **^−5^**	↓ (−0.27)	Artery—Aorta
	*C19orf53*	**8.9 × 10** **^−18^**	↓ (−0.17)	Artery—Tibial
	*MRI1*	**1.0 × 10** **^−5^**	↓ (−0.23)	Artery—Tibial
	*C19orf53*	**9.4 × 10** **^−7^**	↓ (−0.33)	Brain—Amygdala
	*C19orf53*	**5.1 × 10** **^−8^**	↓ (−0.34)	Brain—Anterior cingulate cortex (BA24)
	*C19orf53*	**2.5 × 10** **^−18^**	↓ (−0.36)	Brain—Caudate (basal ganglia)
	*C19orf53*	**2.8 × 10** **^−6^**	↓ (−0.27)	Brain—Cerebellum
	*C19orf53*	**4.9 × 10** **^−15^**	↓ (−0.42)	Brain—Cortex
	*MRI1*	**1.7 × 10** **^−5^**	↓ (−0.39)	Brain—Cortex
	*C19orf53*	**1.2 × 10** **^−15^**	↓ (−0.38)	Brain—Frontal Cortex (BA9)
	*C19orf53*	**6.2 × 10** **^−12^**	↓ (−0.31)	Brain—Hippocampus
	*C19orf53*	**3.6 × 10** **^−7^**	↓ (−0.24)	Brain—Hypothalamus
	*C19orf53*	**3.0 × 10** **^−15^**	↓ (−0.33)	Brain—Nucleus accumbens (basal ganglia)
	*C19orf53*	**1.3 × 10** **^−8^**	↓ (−0.3)	Brain—Putamen (basal ganglia)
	*C19orf53*	**6.4 × 10** **^−12^**	↓ (−0.29)	Pituitary
	*C19orf53*	**2.5 × 10** **^−11^**	↓ (−0.15)	Adipose—Subcutaneous
	*MRI1*	**9.** **1 × 10** **^−6^**	↓ (−0.21)	Adipose—Subcutaneous
	*C19orf53*	**2.3 × 10** **^−9^**	↓ (−0.15)	Adipose—Visceral (Omentum)
rs11666524 *C19orf53* (G/**A**)	*C19orf53*	**5.5 × 10** **^−18^**	↓ (−0.23)	Artery—Aorta
	*MRI1*	**2.** **1 × 10** **^−5^**	↓ (−0.27)	Artery—Aorta
	*C19orf53*	**1.7 × 10** **^−17^**	↓ (−0.17)	Artery—Tibial
	*MRI1*	**9.3 × 10** **^−6^**	↓ (−0.23)	Artery—Tibial
	*C19orf53*	**9.3 × 10** **^−7^**	↓ (−0.33)	Brain—Amygdala
	*C19orf53*	**5.6 × 10** **^−8^**	↓ (−0.34)	Brain—Anterior cingulate cortex (BA24)
	*C19orf53*	**7.8 × 10** **^−19^**	↓ (−0.36)	Brain—Caudate (basal ganglia)
	*C19orf53*	**2.7 × 10** **^−6^**	↓ (−0.27)	Brain—Cerebellum
	*C19orf53*	**8.7 × 10** **^−16^**	↓ (−0.42)	Brain—Cortex
	*MRI1*	**2.8 × 10** **^−5^**	↓ (−0.38)	Brain—Cortex
	*C19orf53*	**1.0 × 10** **^−15^**	↓ (−0.37)	Brain—Frontal Cortex (BA9)
	*C19orf53*	**1.4 × 10** **^−10^**	↓ (−0.3)	Brain—Hippocampus
	*C19orf53*	**4.3 × 10** **^−7^**	↓ (−0.23)	Brain—Hypothalamus
	*C19orf53*	**1.9 × 10** **^−14^**	↓ (−0.32)	Brain—Nucleus accumbens (basal ganglia)
	*C19orf53*	**8.6 × 10** **^−9^**	↓ (−0.3)	Brain—Putamen (basal ganglia)
	*C19orf53*	**8.5 × 10** **^−12^**	↓ (−0.29)	Pituitary
	*C19orf53*	**1.6 × 10** **^−11^**	↓ (−0.16)	Adipose—Subcutaneous
	*MRI1*	**6.** **1 × 10** **^−6^**	↓ (−0.21)	Adipose—Subcutaneous
	*C19orf53*	**5.1 × 10** **^−9^**	↓ (−0.14)	Adipose—Visceral (Omentum)
rs346157 *C19orf53* (A/**G**)	*C19orf53*	**6.2 × 10** **^−10^**	↓ (−0.15)	Artery—Aorta
	*C19orf53*	**3.2 × 10** **^−10^**	↓ (−0.11)	Artery—Tibial
	*C19orf53*	**2.1 × 10** **^−7^**	↓ (−0.27)	Brain—Cortex
	*C19orf53*	**3.9 × 10** **^−6^**	↓ (−0.22)	Brain—Frontal Cortex (BA9)
	*C19orf53*	**2.5 × 10** **^−5^**	↓ (−0.17)	Brain—Nucleus accumbens (basal ganglia)
	*C19orf53*	**5.9 × 10** **^−8^**	↓ (−0.2)	Pituitary
	*C19orf53*	**9.6 × 10** **^−8^**	↓ (−0.11)	Adipose—Subcutaneous
rs346158 *C19orf53* (T/**C**)	*C19orf53*	**9.5 × 10** **^−14^**	↓ (−0.19)	Artery—Aorta
	*MRI1*	**6.3 × 10** **^−5^**	↓ (−0.25)	Artery—Aorta
	*C19orf53*	**4.1 × 10** **^−13^**	↓ (−0.14)	Artery—Tibial
	*MRI1*	**5.2 × 10** **^−5^**	↓ (−0.2)	Artery—Tibial
	*C19orf53*	**1.1 × 10** **^−5^**	↓ (−0.3)	Brain—Amygdala
	*C19orf53*	**1.7 × 10** **^−7^**	↓ (−0.32)	Brain—Anterior cingulate cortex (BA24)
	*C19orf53*	**2.8 × 10** **^−13^**	↓ (−0.31)	Brain—Caudate (basal ganglia)
	*C19orf53*	**5.0 × 10** **^−10^**	↓ (−0.36)	Brain—Cortex
	*MRI1*	**8.9 × 10** **^−7^**	↓ (−0.47)	Brain—Cortex
	*C19orf53*	**3.2 × 10** **^−15^**	↓ (−0.38)	Brain—Frontal Cortex (BA9)
	*C19orf53*	**3.8 × 10** **^−8^**	↓ (−0.26)	Brain—Hippocampus
	*C19orf53*	**7.8 × 10** **^−6^**	↓ (−0.21)	Brain—Hypothalamus
	*C19orf53*	**3.8 × 10** **^−10^**	↓ (−0.28)	Brain—Nucleus accumbens (basal ganglia)
	*C19orf53*	**1.0 × 10** **^−5^**	↓ (−0.25)	Brain—Putamen (basal ganglia)
	*C19orf53*	**8.4 × 10** **^−9^**	↓ (−0.25)	Pituitary
	*C19orf53*	**4.4 × 10** **^−10^**	↓ (−0.14)	Adipose—Subcutaneous
	*MRI1*	**5.9 × 10** **^−7^**	↓ (−0.23)	Adipose—Subcutaneous
	*C19orf53*	**2.4 × 10** **^−7^**	↓ (−0.13)	Adipose—Visceral (Omentum)
rs2277947 *C19orf53* (G/**A**)	*C19orf53*	**5.5 × 10** **^−18^**	↓ (−0.23)	Artery—Aorta
	*MRI1*	**2.1 × 10** **^−5^**	↓ (−0.27)	Artery—Aorta
	*C19orf53*	**1.7 × 10** **^−17^**	↓ (−0.17)	Artery—Tibial
	*MRI1*	**9.3 × 10** **^−6^**	↓ (−0.23)	Artery—Tibial
	*C19orf53*	**9.3 × 10** **^−7^**	↓ (−0.33)	Brain—Amygdala
	*C19orf53*	**9.9 × 10** **^−8^**	↓ (−0.34)	Brain—Anterior cingulate cortex (BA24)
	*C19orf53*	**5.2 × 10** **^−18^**	↓ (−0.36)	Brain—Caudate (basal ganglia)
	*C19orf53*	**3.0 × 10** **^−6^**	↓ (−0.27)	Brain—Cerebellum
	*C19orf53*	**1.1 × 10** **^−14^**	↓ (−0.41)	Brain—Cortex
	*MRI1*	**1.0 × 10** **^−5^**	↓ (−0.4)	Brain—Cortex
	*C19orf53*	**4.7 × 10** **^−15^**	↓ (−0.37)	Brain—Frontal Cortex (BA9)
	*C19orf53*	**1.4 × 10** **^−10^**	↓ (−0.3)	Brain—Hippocampus
	*C19orf53*	**4.3 × 10** **^−7^**	↓ (−0.23)	Brain—Hypothalamus
	*C19orf53*	**1.9 × 10** **^−14^**	↓ (−0.32)	Brain—Nucleus accumbens (basal ganglia)
	*C19orf53*	**2.4 × 10** **^−8^**	↓ (−0.3)	Brain—Putamen (basal ganglia)
	*C19orf53*	**8.5 × 10** **^−12^**	↓ (−0.29)	Pituitary
	*C19orf53*	**4.8 × 10** **^−11^**	↓ (−0.15)	Adipose—Subcutaneous
	*MRI1*	**5.** **1 × 10** **^−6^**	↓ (−0.21)	Adipose—Subcutaneous
	*C19orf53*	**6.2 × 10** **^−9^**	↓ (−0.14)	Adipose—Visceral (Omentum)

Effect alleles are marked in bold.

**Table 5:** The effect of *C19orf53* SNPs on histone modifications in different tissues.

SNP (Ref/Alt Allele)	Tissue Marks	Brain Regions							Peripheral Blood	Adipose
		(1)	(2)	(3)	(4)	(5)	(6)	(7)	(8)	(9)
rs10104 *C19orf53* (A/**G**)	H3K4me1	Enh	Enh	Enh	Enh	-	Enh	Enh	Enh	Enh
	H3K4me3	Pro	Pro	Pro	Pro	Pro	Pro	Pro	Pro	Pro
	H3K27ac	Enh	Enh	Enh	Enh	Enh	Enh	Enh	Enh	Enh
	H3K9ac	-	Pro	Pro	Pro	Pro	Pro	Pro	Pro	Pro
	DNase	-	-	-	-	-	-	-	DNase	-
rs11666524 *C19orf53* (G/**A**)	H3K4me1	Enh	Enh	Enh	Enh	Enh	Enh	Enh	Enh	Enh
	H3K4me3	Pro	Pro	Pro	Pro	-	-	-	Pro	Pro
	H3K27ac	Enh	Enh	Enh	Enh	Enh	-	-	Enh	Enh
	H3K9ac	-	Pro	Pro	Pro	-	Pro	Pro	Pro	Pro
	DNase	-	-	-	-	-	-	-	-	-
rs346157 *C19orf53* (A/**G**)	H3K4me1	-	Enh	Enh	-	Enh	Enh	Enh	Enh	-
	H3K4me3	-	-	-	-	-	-	-	Pro	Pro
	H3K27ac	Enh	Enh	Enh	Enh	Enh	-	-	Enh	Enh
	H3K9ac	-	Pro	-	Pro	-	-	-	Pro	-
	DNase	-	-	-	-	-	-	-	-	-
rs346158 *C19orf53* (T/**C**)	H3K4me1	-	-	Enh	-	-	Enh	Enh	Enh	-
	H3K4me3	-	-	-	-	-	-	-	Pro	Pro
	H3K27ac	Enh	Enh	Enh	Enh	Enh	-	Enh	Enh	Enh
	H3K9ac	-	Pro	-	Pro	-	-	-	Pro	-
	DNase	-	-	-	-	-	-	-	-	-
rs2277947 *C19orf53* (G/**A**)	H3K4me1	Enh	Enh	Enh	Enh	Enh	-	Enh	Enh	Enh
	H3K4me3	-	-	-	-	-	-	-	Pro	Pro
	H3K27ac	-	-	-	-	-	-	-	Enh	Enh
	H3K9ac	-	-	-	-	-	-	-	Pro	Pro
	DNase	-	-	-	-	-	-	-	-	-

H3K4me1—mono-methylation at the 4th lysine residue of the histone H3 protein; H3K4me3—tri-methylation at the 4th lysine residue of the histone H3 protein; H3K9ac—acetylation at the 9th lysine residues of the histone H3 protein; H3K27ac—acetylation of the lysine residue at N-terminal position 27 of the histone H3 protein; effect alleles are marked in bold. Enh—histone modification associated with gene enhancers; Pro—histone modification associated with gene promoters; 1—Brain (hippocampus); 2—Brain (substantia nigra); 3—Brain (anterior caudate); 4—Brain (cingulate gyrus); 5—Brain (inferior temporal lobe); 6—Brain (angular gyrus); 7—Brain (dorsolateral prefrontal cortex); 8—Any cells from peripheral blood; 9—Adipose Nuclei.

**Table 6:** Results of bioinformatic analyses for *C19orf53* SNPs using Cerebrovascular and Cardiovascular Disease Knowledge Portals.

Tag SNPs	Phenotype	*p*-Value	Beta (OR)	Sample Size
rs10104 *C19orf53* (A/**G**)	^1^ All ischemic stroke	0.015	_OR_▲1.0173	1,047,650
	^1^ Modified Rankin scale score 0–2 vs. 3–6 adj stroke severity	0.04	_OR_▲1.1224	5666
	^2^ Atrial fibrillation or flutter	0.014	_OR_▲1.0291	130,776
	^2^ Triglyceride-to-HDL ratio	0.023	_Beta_▲0.0058	579,558
	^2^ Serum ApoB	0.028	_Beta_▼−0.0048	450,527
rs11666524 *C19orf53* (G/**A**)	^1^ All ischemic stroke	0.0004	_OR_▲1.0214	794,817
	^1^ Modified Rankin scale score 0–2 vs. 3–6 adj stroke severity	0.029	_OR_▲1.1324	5666
	^1^ Any stroke	0.046	_OR_▲1.0178	851,850
	^2^ Atrial fibrillation or flutter	0.01	_OR_▲1.0293	130,776
	^2^ Triglyceride-to-HDL ratio	0.029	_Beta_▲0.0075	418,488
	^2^ Serum ApoB	0.03	_Beta_▼−0.0047	436,068
rs346157 *C19orf53* (A/**G**)	^1^ All ischemic stroke	0.005	_OR_▲1.0707	38,850
	^1^ Cerebral white matter hyperintensity volume	0.033	_Beta_▲0.0267	11,226
	^2^ Dyslipidemia	0.01	_OR_▲1.0360	56,375
	^2^ Obese vs. controls	0.040	_OR_▲1.0896	4752
rs346158 *C19orf53* (T/**C**)	^1^ Any stroke	0.006	_OR_▲1.0870	12,406
	^1^ All ischemic stroke	0.003	_OR_▲1.0202	1,042,380
	^1^ Modified Rankin scale score 0–2 vs. 3–6 adj stroke severity	0.012	_OR_▲1.1511	5666
	^1^ Modified Rankin scale score 0–2 vs. 3–6	0.04	_OR_▲1.1030	5802
	^2^ Triglyceride-to-HDL ratio	0.01	_Beta_▲0.0085	418,488
	^2^ Atrial fibrillation or flutter	0.015	_OR_▲1.0289	130,776
	^2^ Serum ApoB	0.04	_Beta_▼−0.0045	436,068
rs2277947 *C19orf53* (G/**A**)	^1^ All ischemic stroke	0.005	_OR_▲1.0193	1,062,920
	^1^ Modified Rankin scale score 0–2 vs. 3–6 adj stroke severity	0.03	_OR_▲1.1308	5666
	^2^ Atrial fibrillation or flutter	0.01	_OR_▲1.0292	130,776
	^2^ Triglyceride-to-HDL ratio	0.02	_Beta_▲0.0079	418,488
	^2^ Serum ApoB	0.035	_Beta_▼−0.0047	436,068

^1^—Cerebrovascular Disease Knowledge Portal data; ^2^—Cardiovascular Disease Knowledge Portal data. Effect alleles are marked in bold.

## Data Availability

The data presented in this study are available upon request from corresponding author.

## Supplementary Materials

The following supporting information can be downloaded at https://www.mdpi.com/article/10.3390/life14091158/s1, Supplementary Figure S1: Allelic discrimination plots for *C19orf53* assays designed for this study. (A) rs10104 *C19orf53.* (B) rs11666524 *C19orf53.* (C) rs346157 *C19orf53.* (D) rs346158 *C19orf53.* (E) rs2277947 *C19orf53.* (F) rs2901077 *C19orf53.* (G) rs8107914 *C19orf53;* Supplementary Figure S2: *C19orf53* expression levels in vessels, brain, and peripheral blood; Supplementary Figure S3: Predicted interaction partners of *C19orf53*; Table S1: Primers and probes designed for the study; Table S2: The analysis of the correspondence of the distribution of *C19orf53* genotypes to the Hardy–Weinberg equilibrium; Table S3: The relationship between *C19orf53* gene SNPs and ischemic stroke risk (entire group); Table S4: Smoking, BMI, fruit and vegetable intake, and physical activity level-depending associations between *C19orf53* SNPs and IS risk; Table S5: Statistically significant correlations between *C19orf53* SNPs and clinical parameter IS patients; Table S6: Mechanisms of interactions between *C19orf53* and cis-eQTL-related genes (results of analysis using the GeneMania resource); Table S7: Biological processes characterizing the joint functions of genes in a network of interactions of *C19orf53* and cis-eQTL-related genes; Table S8: Effects of rs10104 *C19orf53* on the binding of DNA to TFs; Table S9: Effects of rs11666524 *C19orf53* on the binding of DNA to TFs; Table S10: Effects of rs346157 *C19orf53* on the binding of DNA to TFs; Table S11: Effects of rs2277947 *C19orf53* on the binding of DNA to TFs; Table S12: Effects of rs346158 *C19orf53* on the binding of DNA to TFs; Table S13: Basic molecular characteristics of predicted functional partners of *C19orf53;* Table S14: Functional enrichments of C19orf53 network.
