# Supplementary material for: Obesity and Environmental Risk Factors Significantly Modify the Association between Ischemic Stroke and the Hero Chaperone C19orf53
Source: Life (Basel). 2024 Sep 12;14(9):1158. doi: 10.3390/life14091158 (PMC11433390; doi:10.3390/life14091158)
Supplement: Supplementary file 1 [file life-14-01158-s001.zip › life-3160444-supplementary.pdf]

Supplementary materials

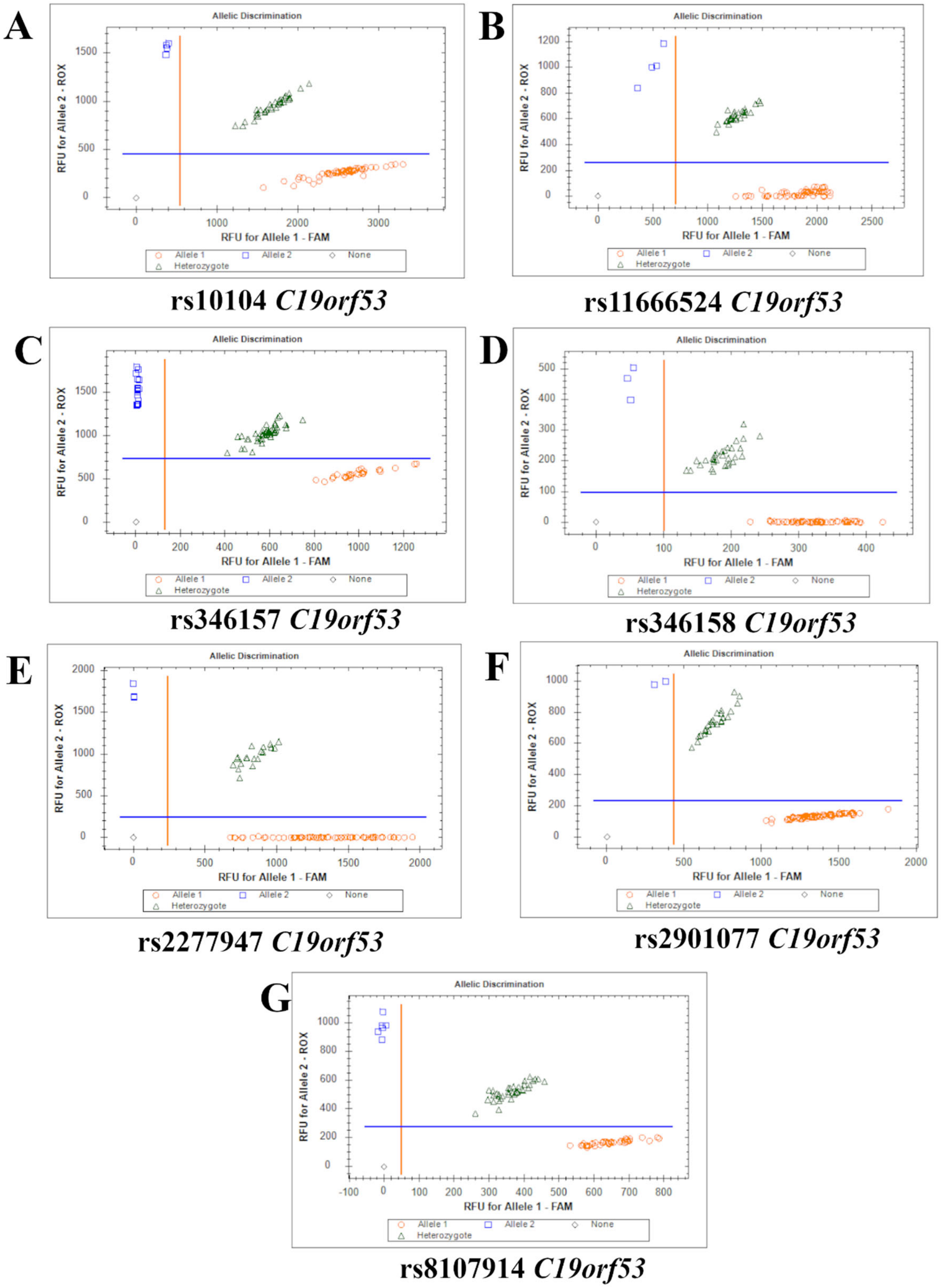

Supplementary Figure 1: Allelic discrimination plots for *C19orf53* assays designed for this study. (A) rs10104 *C19orf53*. (B) rs11666524 *C19orf53*. (C) rs346157 *C19orf53*. (D) rs346158 *C19orf53*. (E) rs2277947 *C19orf53*. (F) rs2901077 *C19orf53*. (G) rs8107914 *C19orf53*.

Table S1: Primers and probes designed for the study

rs10104 *C19orf53*	5′- TCACGTGAGCACATCTTTCC-3′<br>5′- CTCCGGGCTCCATCTCTC -3′<br>5′- FAM-TCGCTCCCAAGAAGGCGC-RTQ1-3′<br>5′- ROX-TCGCTCCCAGGAAGGCGC-BHQ2-3′
rs11666524 *C19orf53*	5′- GCCCCTATTTTGCCCTTAGA-3′<br>5′- GGCTTCTGTGAGAGGACCAG-3′<br>5′-FAM- TCCCACCGGGACCAGTGC-RTQ1-3′<br>5′-ROX- TCCCACCAGGACCAGTGC-BHQ2-3′
rs346157 *C19orf53*	5′-CAGCACACCGTGCTAATTCT-3′<br>5′-CCCTGCCATTCTGAGTTCC-3′<br>5′-FAM-CTTCCTGCCTCAGCCTCGCA-RTQ1-3′<br>5′-ROX-CTTCCTGCCTCGGCCTCGCA-BHQ2-3′
rs346158 *C19orf53*	5′- AGTTTGGGGTTCACACTGCT -3′<br>5′- CCTCTGGTCCCAGCCTCTA -3′<br>5′-FAM-TGCCGTCTGCAAAGCA-RTQ1-3′<br>5′-ROX-TGCCGTCCGCAAAGCA-BHQ2-3′
rs2277947 *C19orf53*	5′- ATCCCTCTTGCAGTCCTGTG -3′<br>5′- AAGACTCTTTCCCGGCTCTC -3′<br>5′-FAM-CACCCCACCGCCTTTAGCA-RTQ1-3′<br>5′-ROX-CACCCCACCACCTTTAGCA-BHQ2-3′
rs2901077 *C19orf53*	5′- TGCAAAGCAGAGATGACCTG-3′<br>5′- CCATTTTATGGATGGGGAAA-3′<br>5′- FAM- CACGCCGGATCCCATGC-RTQ1-3′<br>5′- ROX- CACGCTGGATCCCATGC-BHQ2-3′
rs8107914 *C19orf53*	5′- CGCCTTTAGCAACCATGTG -3′<br>5′- CCAGCTTGACTCTGGTTGTG-3′<br>5′- FAM- CCGGGAAAGAGTCTTTTCTCC-RTQ1-3′<br>5′- ROX- CCGGGAAAGAGTTTTTTCTCC-BHQ2-3′

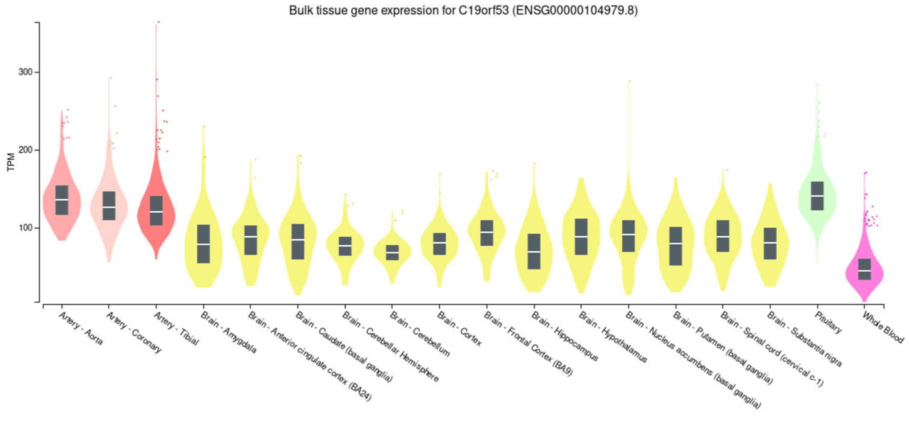

Supplementary Figure 2: *C19orf53* expression levels in vessels, brain, and peripheral blood

Table S2: Analysis of the correspondence of the distribution of *C19orf53* genotypes to the Hardy-Weinberg equilibrium

SNP	Genotypes	Controls	H_o (H_e)[1]	P[2]	IS patients	H_o (H_e)[3]	P[4]
rs10104	A/A	693 (57.8%)	0.38 (0.36)	>0.05	532 (60.4%)	0.33 (0.35)	>0.05
	A/G	453 (37.8%)			293 (33.3%)		
	G/G	53 (4.4%)			56 (6.4%)		
	Maf(G)	0.233			0.230		
rs11666524	G/G	710 (58.1%)	0.37 (0.36)	>0.05	534 (60.1%)	0.33 (0.35)	0.01
	G/A	453 (37.1%)			291 (32.8%)		
	A/A	59 (4.8%)			63 (7.1%)		
	Maf(A)	0.234			0.235		
rs346157	A/A	430 (34.7%)	0.50 (0.48)	>0.05	316 (35.8%)	0.45 (0.49)	0.031
	A/G	616 (49.8%)			398 (45%)		
	G/G	192 (15.5%)			170 (19.2%)		
	Maf(G)	0.404			0.417		
rs346158	T/T	705 (57.6%)	0.37 (0.36)	>0.05	532 (60%)	0.32 (0.36)	0.004
	T/C	457 (37.3%)			288 (32.5%)		
	C/C	62 (5.1%)			67 (7.5%)		
	Maf(C)	0.237			0.238		
rs2901077	C/C	964 (80.2%)	0.19 (0.19)	>0.05	686 (78.6%)	0.19 (0.21)	>0.05
	C/T	228 (19%)			170 (19.5%)		
	T/T	10 (0.8%)			17 (2%)		
	Maf(T)	0.103			0.117		
rs2277947	G/G	688 (58.8%)	0.36 (0.35)	>0.05	515 (59.9%)	0.33 (0.36)	0.008
	G/A	425 (36.3%)			282 (32.8%)		
	A/A	57 (4.9%)			63 (7.3%)		
	Maf(A)	0.230			0.237		
rs8107914	C/C	701 (58.1%)	0.36 (0.36)	>0.05	532 (60.7%)	0.32 (0.36)	0.032
	C/T	432 (35.8%)			283 (32.3%)		
	T/T	73 (6%)			61 (7%)		
	Maf(T)	0.240			0.231		

[1] – observed (Ho) and expected (He) heterozygosity in healthy controls; [2] – P-HWE in healthy controls; [3] – observed (Ho) and expected (He) heterozygosity in IS patients; [4] – P-HWE in IS patients

Table S3: The relationship between C19orf53 gene SNPs and ischemic stroke risk (entire group)

SNP	N	Dominant model		Recessive model		Log-additive model	
		OR (95% CI)[1]	P (P_{bonf})	OR (95% CI)[1]	P (P_{bonf})	OR (95% CI)[1]	P (P_{bonf})
rs10104 *C19orf53* (A/G)	2080	0.87 [0.73-1.05]	0.14 (0.4)	1.43 [0.96-2.13]	0.081 (0.24)	0.96 [0.82-1.11]	0.58 (1.7)
rs11666524 *C19orf53* (G/A)	2110	0.89 [0.74-1.07]	0.21 (0.6)	1.40 [0.96-2.05]	0.078 (0.23)	0.97 [0.84-1.13]	0.73 (1.0)
rs346157 *C19orf53* (A/G)	2122	0.95 [0.79-1.15]	0.6 (1.0)	**1.30 [1.03-1.64]**	**0.03** (0.09)	1.05 [0.93-1.20]	0.42 (1.0)
rs346158 *C19orf53* (T/C)	2111	0.88 [0.73-1.05]	0.16 (0.5)	**1.46 [1.01-2.11]**	**0.04** (0.12)	0.97 [0.84-1.13]	0.73 (1.0)
rs2901077	2075	1.11	0.35	**2.49**	**0.02**	1.16	0.15

C19orf53 (C/T)		[0.89-1.39]	(1.0)	**[1.11-5.57]**	(0.06)	[0.95-1.42]	(0.5)
rs2277947 *C19orf53* (G/A)	2030	0.92 [0.77-1.11]	0.41 (1.0)	1.44 [0.99-2.11]	0.06 (0.18)	1.01 [0.87-1.17]	0.94 (1.0)
rs8107914 *C19orf53* (C/T)	2082	0.87 [0.73-1.05]	0.14 (0.1)	1.13 [0.79-1.63]	0.5 (1.0)	0.94 [0.81-1.08]	0.37 (1.0)
All calculations were performed relative to the minor alleles (Effect allele) with adjustment for sex, age, smoking; 1 - odds ratio and 95% confidence interval; 2– P- value; statistically significant differences are marked in bold. Effect alleles are marked in bold							

Table S4: Smoking , BMI, fruit and vegetable intake, physical activity levels-depending associations between *C19orf53* SNPs and IS risk

SNP	N	Dominant model		Recessive model		Log-additive model		N	Dominant model		Recessive model		Log-additive model	
		OR (95% CI)[1]	P (P_{bonf})	OR (95% CI)[1]	P (P_{bonf})	OR (95% CI)[1]	P (P_{bonf})		OR (95% CI)[1]	P (P_{bonf})	OR (95% CI)[1]	P (P_{bonf})	OR (95% CI)[1]	P (P_{bonf})
	Smokers							Non-smokers						
rs10104 *C19orf53* (A/G)	850	0.84 [0.64-1.11]	0.21	1.16 [0.66-2.05]	0.6	0.91 [0.73-1.14]	0.42	1326	0.93 [0.74-1.18]	0.56	1.61 [0.96-2.70]	0.072	1.02 [0.84-1.23]	0.86
rs11666524 *C19orf53* (G/A)	865	0.89 [0.68-1.17]	0.4	1.26 [0.74-2.14]	0.39	0.97 [0.78-1.20]	0.76	1343	0.92 [0.73-1.16]	0.48	1.53 [0.93-2.53]	0.1	1.00 [0.83-1.21]	0.98
rs346157 *C19orf53* (A/G)	874	1.06 [0.81-1.40]	0.67	1.17 [0.82-1.66]	0.39	1.07 [0.89-1.29]	0.45	1346	0.91 [0.72-1.15]	0.44	1.34 [1.00-1.80]	0.054	1.04 [0.89-1.23]	0.6
rs346158 *C19orf53* (T/C)	873	0.82 [0.63-1.08]	0.16	1.20 [0.70-2.04]	0.51	0.91 [0.73-1.13]	0.39	1333	0.93 [0.74-1.17]	0.54	1.64 [1.01-2.64]	0.046	1.03 [0.85-1.24]	0.79
rs2901077 *C19orf53* (C/T)	848	1.17 [0.83-1.64]	0.37	1.96 [0.60-6.42]	0.25	1.19 [0.88-1.61]	0.26	1324	1.10 [0.83-1.45]	0.51	2.11 [0.81-5.50]	0.13	1.14 [0.88-1.46]	0.32
rs2277947 *C19orf53* (G/A)	830	0.92 [0.70-1.22]	0.56	1.46 [0.84-2.55]	0.17	1.01 [0.81-1.26]	0.93	1287	0.99 [0.78-1.25]	0.92	1.48 [0.90-2.44]	0.13	1.05 [0.87-1.27]	0.63
rs8107914 *C19orf53* (C/T)	852	0.87 [0.66-1.15]	0.33	1.22 [0.71-2.09]	0.48	0.95 [0.76-1.18]	0.62	1324	0.90 [0.72-1.14]	0.38	1.10 [0.69-1.75]	0.7	0.95 [0.79-1.14]	0.58
	BMI≥30							BMI<30						
rs10104 *C19orf53* (A/G)	1286	1.52 [0.90-2.55]	0.12 (0.36)	**4.70 [2.25-9.82]**	**0.0003 (0.0009)**	**1.82 [1.22-2.72]**	**0.004 (0.01)**	1745	0.94 [0.76-1.16]	0.57 (1.0)	1.60 [1.02-2.51]	0.046 (1.0)	1.02 [0.86-1.22]	0.79 (1.0)
rs11666524	1330	1.54 [0.92-2.58]	0.1 (0.3)	**3.88 [1.88-8.02]**	**0.001 (0.003)**	**1.76 [1.19-2.60]**	**0.006 (0.02)**	1792	0.94 [0.76-1.15]	0.55 (1.0)	1.64 [1.09-2.48]	0.02 (0.06)	1.04 [0.88-1.23]	0.68 (1.0)

C19orf53 (G/A)														
rs346157 *C19orf53* (A/G)	1315	1.04 [0.60-1.80]	0.9 (1.0)	1.50 [0.80-2.83]	0.23 (0.69)	1.16 [0.79-1.69]	0.45 (1.0)	1780	0.92 [0.74-1.14]	0.44 (1.0)	1.27 [0.97-1.65]	0.08 (1.0)	1.03 [0.89-1.19]	0.68 (1.0)
rs346158 *C19orf53* (T/C)	1301	1.40 [0.84-2.35]	0.2 (0.6)	**3.73 [1.81-7.69]**	**0.002 (0.006)**	**1.65 [1.11-2.44]**	**0.015 (0.045)**	1769	0.90 [0.73-1.11]	0.32 (0.96)	1.60 [1.06-2.40]	0.03 (0.09)	1.01 [0.85-1.19]	0.94 (1.0)
rs2901077 *C19orf53* (C/T)	1337	1.04 [0.54-1.99]	0.91 (1.0)	3.70 [0.81-16.93]	0.15 (0.45)	1.17 [0.66-2.08]	0.6 (1.0)	1795	1.08 [0.83-1.38]	0.58 (1.0)	1.66 [0.67-4.08]	0.28 (0.84)	1.10 [0.87-1.38]	0.43 (1.0)
rs2277947 *C19orf53* (G/A)	1242	1.60 [0.95-2.70]	0.08 (0.24)	**4.11 [1.98-8.53]**	**0.0008 (0.002)**	**1.82 [1.22-2.70]**	**0.004 (0.01)**	1699	0.98 [0.80-1.21]	0.87 (1.0)	1.60 [1.05-2.45]	0.03 (0.09)	1.06 [0.90-1.26]	0.48 (1.0)
rs8107914 *C19orf53* (C/T)	1341	1.35 [0.80-2.28]	0.26 (0.78)	2.52 [1.16-5.51]	0.035 (1.0)	1.45 [0.98-2.16]	0.069 (1.0)	1799	0.94 [0.76-1.15]	0.53 (1.0)	1.20 [0.80-1.82]	0.38 (1.0)	0.99 [0.83-1.17]	0.87 (1.0)
	Low fruit/vegetable intake (f+)							Normal fruit/vegetable intake (f-)						
rs10104 *C19orf53* (A/G)	1712	0.98 [0.79-1.22]	0.87 (2.6)	1.62 [1.03-2.54]	0.039 (0.112)	1.06 [0.89-1.27]	0.52 (1.6)	1659	0.82 [0.65-1.03]	0.08 (0.2)	1.24 [0.75-2.06]	0.41 (1.2)	0.89 [0.73-1.08]	0.24 (0.7)
rs11666524 *C19orf53* (G/A)	1739	0.97 [0.78-1.21]	0.81 (2.4)	**1.81 [1.20-2.75]**	**0.006 (0.02)**	1.08 [0.91-1.29]	0.37 (1.1)	1682	0.86 [0.69-1.09]	0.21 (0.6)	1.13 [0.68-1.86]	0.64 (1.9)	0.92 [0.76-1.11]	0.38 (1.1)
rs346157 *C19orf53* (A/G)	1752	0.91 [0.73-1.13]	0.4 (1.2)	**1.42 [1.08-1.86]**	**0.012 (0.036)**	1.06 [0.91-1.24]	0.43 (1.3)	1699	1.04 [0.82-1.31]	0.76 (2.3)	1.14 [0.85-1.53]	0.4 (1.2)	1.06 [0.90-1.24]	0.51 (1.5)
rs346158 *C19orf53* (T/C)	1740	0.96 [0.77-1.19]	0.71 (2.1)	**1.93 [1.29-2.89]**	**0.0017 (0.005)**	1.09 [0.92-1.30]	0.33 (1.0)	1687	0.86 [0.68-1.08]	0.18 (0.5)	1.07 [0.65-1.76]	0.79 (2.4)	0.91 [0.75-1.10]	0.31 (0.9)
rs2901077 *C19orf53* (C/T)	1709	1.15 [0.89-1.50]	0.29 (0.9)	2.32 [0.99-5.40]	0.058 (0.17)	1.19 [0.94-1.51]	0.14 (0.4)	1656	1.04 [0.78-1.37]	0.81 (2.4)	1.56 [0.58-4.19]	0.39 (1.2)	1.06 [0.82-1.37]	0.66 (2.0)

rs2277947<br>*C19orf53*<br>(G/A)	1668	1.02<br>[0.82-1.27]	0.86<br>(2.6)	**1.81<br>[1.18-2.76]**	**0.0074<br>(0.02)**	1.12<br>[0.93-1.33]	0.23<br>(0.7)	160<br>4	0.90<br>[0.71-1.14]	0.38<br>(1.1)	1.24<br>[0.75-2.04]	0.4<br>(1.2)	0.96<br>[0.79-1.17]	0.68<br>(2.0)
rs8107914<br>*C19orf53*<br>(C/T)	1716	0.97<br>[0.78-1.21]	0.79<br>(2.4)	1.46<br>[0.98-2.19]	0.43<br>(1.29)	1.05<br>[0.88-1.24]	0.59<br>(1.8)	166<br>0	0.82<br>[0.65-1.03]	0.09<br>(0.3)	0.82<br>[0.50-1.37]	0.44<br>(1.3)	0.85<br>[0.70-1.03]	0.09<br>(0.3)
	Low physical activity level (f+)							Normal physical activity level (f-)						
rs10104<br>*C19orf53*<br>(A/G)	1597	0.86<br>[0.67-1.09]	0.21<br>(0.63)	**1.91<br>[1.19-3.07]**	**0.009<br>(0.03)**	1.00<br>[0.82-1.22]	0.99<br>(2.3)	177<br>4	0.93<br>[0.76-1.15]	0.5<br>(1.5)	1.14<br>[0.71-1.83]	0.6<br>(1.8)	0.97<br>[0.81-1.15]	0.7<br>(2.1)
rs11666524<br>*C19orf53*<br>(G/A)	1623	0.85<br>[0.67-1.08]	0.18<br>(0.5)	**1.80<br>[1.13-2.84]**	**0.016<br>(0.048)**	0.99<br>[0.81-1.20]	0.91<br>(2.7)	179<br>8	0.97<br>[0.79-1.20]	0.79<br>(2.4)	1.29<br>[0.83-2.00]	0.26<br>(0.8)	1.02<br>[0.86-1.21]	0.84<br>(2.5)
rs346157<br>*C19orf53*<br>(A/G)	1637	0.88<br>[0.69-1.13]	0.31<br>(0.9)	1.20<br>[0.88-1.64]	0.25<br>(0.75)	0.99<br>[0.84-1.18]	0.93<br>(2.8)	181<br>4	1.03<br>[0.83-1.27]	0.79<br>(2.4)	1.34<br>[1.03-1.74]	0.03<br>(0.1)	1.11<br>[0.96-1.28]	0.17<br>(0.5)
rs346158<br>*C19orf53*<br>(T/C)	1625	0.83<br>[0.65-1.06]	0.14<br>(0.4)	1.72<br>[1.09-2.71]	0.024<br>(0.06)	0.97<br>[0.80-1.18]	0.77<br>(2.3)	180<br>2	0.96<br>[0.78-1.18]	0.72<br>(2.2)	1.39<br>[0.91-2.12]	0.13<br>(0.4)	1.03<br>[0.87-1.21]	0.77<br>(2.3)
rs2901077<br>*C19orf53*<br>(C/T)	1594	1.20<br>[0.90-1.60]	0.22<br>(0.7)	2.17<br>[0.85-5.55]	0.12<br>(0.36)	1.23<br>[0.95-1.60]	0.13<br>(0.4)	177<br>1	1.03<br>[0.80-1.33]	0.82<br>(2.5)	1.83<br>[0.77-4.37]	0.18<br>(0.5)	1.07<br>[0.85-1.35]	0.58<br>(1.7)
rs2277947<br>*C19orf53*<br>(G/A)	1551	0.90<br>[0.71-1.16]	0.42<br>(1.3)	1.79<br>[1.12-2.86]	0.018<br>(0.054)	1.03<br>[0.84-1.26]	0.77<br>(2.3)	172<br>1	1.00<br>[0.81-1.24]	0.97<br>(2.9)	1.38<br>[0.89-2.14]	0.16(0<br>.5)	1.05<br>[0.88-1.25]	0.57<br>(1.7)
rs8107914<br>*C19orf53*<br>(C/T)	1598	0.81<br>[0.63-1.04]	0.092<br>(0.3)	1.46<br>[0.94-2.29]	0.1<br>(0.3)	0.94<br>[0.77-1.14]	0.51<br>(1.5)	177<br>8	0.96<br>[0.78-1.18]	0.7<br>(2.1)	0.96<br>[0.62-1.49]	0.86<br>(2.6)	0.97<br>[0.82-1.15]	0.7<br>(2.1)
All calculations were performed relative to the minor alleles (Effect allele); 1 - odds ratio and 95% confidence interval; 2– P- value; statistically significant differences are marked in bold. Effect alleles are marked in bold														

Table S5: Statistically significant correlations between *C19orf53* SNPs and clinical parameters IS patients

SNP	Group	Genotypes	N	Me [Q1-Q3]	M–W U test:
APTT					
rs346157	Low fruit/ vegetable intake	A/A-A/G	197	33 [30.9-37]	**0.03**
		G/G	45	31.3 [28-35]	
BMI					
rs2277947	Nonsmokers	G/G-G/A	249	23 [22-26]	**0.01**
		A/A	19	28 [23-30]	
	Low fruit/ vegetable intake	G/G-G/A	217	23.4 [22-26]	**0.004**
		A/A	23	28 [23-31]	

Table S6: Mechanisms of interactions between C19orf53 and cis-eQTL-related genes (results of analysis using the GeneMania resource)

Gene 1	Gene 2	Weight	Network group	Network
EIF2B1	CCDC130	0.013359001	Co-expression	Wang-Maris-2006
NUP133	TOP2B	0.012292383	Co-expression	Wang-Maris-2006
YJU2	CCDC130	0.005012826	Co-expression	Roth-Zlotnik-2006
RPE	TOP2B	0.008694904	Co-expression	Roth-Zlotnik-2006
MEMO1	MRI1	0.007161244	Co-expression	Ramaswamy-Golub-2001
JUP	TOP2B	0.011643774	Co-expression	Ramaswamy-Golub-2001
NUP133	MEMO1	0.0084729055	Co-expression	Ramaswamy-Golub-2001
ELOB	C19orf53	0.0075211558	Co-expression	Ramaswamy-Golub-2001
MEMO1	APIP	0.003384038	Co-expression	Innocenti-Brown-2011
NOB1	EIF2B4	0.009241889	Co-expression	Innocenti-Brown-2011
ELOB	C19orf53	0.023960046	Co-expression	Innocenti-Brown-2011
TOP2B	APIP	0.007745941	Co-expression	Dobbin-Giordano-2005
RPE	EIF2B1	0.021105502	Co-expression	Dobbin-Giordano-2005
RPE	MTAP	0.016025998	Co-expression	Rieger-Chu-2004
YJU2	CCDC130	0.011533561	Co-expression	Bild-Nevins-2006 B
YJU2	CCDC130	0.02362037	Co-expression	Burington-Shaughnessy-2008
NUP133	MTAP	0.008096119	Co-expression	Burington-Shaughnessy-2008
EIF2B1	TOP2B	0.0053739883	Co-expression	Boldrick-Relman-2002
EIF2B4	YJU2	0.016996352	Co-expression	Arijs-Rutgeerts-2009
NOB1	C19orf53	0.011942913	Co-expression	Arijs-Rutgeerts-2009
NOB1	MTAP	0.014439114	Co-expression	Arijs-Rutgeerts-2009
RPE	SNX6	0.014086695	Co-expression	Jiang-de Kok-2017
PNP	EIF2B2	0.004985559	Co-expression	Jiang-de Kok-2017
ELOB	C19orf53	0.012327663	Co-expression	Perou-Botstein-2000
NUP133	SNX6	0.0076605934	Co-expression	Chen-Brown-2002
ELOB	CCDC130	0.013615076	Co-expression	Chen-Brown-2002
EIF2B4	C19orf53	0.011272465	Co-localization	Johnson-Shoemaker-2003
NUP133	EIF2B1	0.015782963	Co-localization	Johnson-Shoemaker-2003
SNX6	APIP	0.0011464754	Genetic Interactions	Lin-Smith-2010

TOP2B	MTAP	0.0029807605	Genetic Interactions	Lin-Smith-2010
EIF2B1	C19orf53	0.004840979	Genetic Interactions	Lin-Smith-2010
RPE	TOP2B	0.0037817203	Genetic Interactions	Lin-Smith-2010
MEMO1	SNX6	0.0012433794	Genetic Interactions	Lin-Smith-2010
ADI1	MTAP	0.0010712576	Genetic Interactions	Lin-Smith-2010
ADI1	EIF2B1	0.0010669715	Genetic Interactions	Lin-Smith-2010
NUP133	TOP2B	0.0035302686	Genetic Interactions	Lin-Smith-2010
NUP133	EIF2B1	0.00096791825	Genetic Interactions	Lin-Smith-2010
NUP133	MEMO1	0.000844271	Genetic Interactions	Lin-Smith-2010
NOB1	MTAP	0.0010439744	Genetic Interactions	Lin-Smith-2010
ELOB	SNX6	0.0027668818	Genetic Interactions	Lin-Smith-2010
ELOB	KIF15	0.0023744323	Genetic Interactions	Lin-Smith-2010
LRRC40	EIF2B1	0.00094951916	Genetic Interactions	Lin-Smith-2010
LRRC40	MEMO1	0.00082822226	Genetic Interactions	Lin-Smith-2010
KDM8	RPE	0.002087226	Genetic Interactions	Lin-Smith-2010
EIF2B1	EIF2B4	0.20621867	Pathway	Wu-Stein-2010
EIF2B2	EIF2B4	0.20621867	Pathway	Wu-Stein-2010
EIF2B2	EIF2B1	0.20621867	Pathway	Wu-Stein-2010
SNX6	MRI1	0.70710677	Pathway	REACTOME
SNX6	MTAP	0.70710677	Pathway	REACTOME
EIF2B1	EIF2B4	0.19066781	Pathway	REACTOME
EIF2B2	EIF2B4	0.19066781	Pathway	REACTOME
EIF2B2	EIF2B1	0.19066781	Pathway	REACTOME
APIP	MRI1	0.5246476	Physical Interactions	IREF-reactome
MTAP	MRI1	0.42203587	Physical Interactions	IREF-reactome
EIF2B1	EIF2B4	0.026302613	Physical Interactions	IREF-reactome
EIF2B2	EIF2B4	0.026302613	Physical Interactions	IREF-reactome
EIF2B2	EIF2B1	0.026302613	Physical Interactions	IREF-reactome
APIP	MRI1	0.5246476	Physical Interactions	Vastrik-Stein-2007

MTAP	MRI1	0.42203587	Physical Interactions	Vastrik-Stein-2007
EIF2B1	EIF2B4	0.026302613	Physical Interactions	Vastrik-Stein-2007
EIF2B2	EIF2B4	0.026302613	Physical Interactions	Vastrik-Stein-2007
EIF2B2	EIF2B1	0.026302613	Physical Interactions	Vastrik-Stein-2007
ELOB	ZSWIM4	0.10499964	Physical Interactions	HΓjttenhain-Krogan-2019
PNP	MTAP	0.07164535	Physical Interactions	Kristensen-Foster-2012
KIF15	MRI1	0.3707892	Physical Interactions	Hein-Mann-2015
EIF2B2	EIF2B4	0.76536685	Physical Interactions	IREF-quickgo
EIF2B1	MTAP	0.03753282	Physical Interactions	Wan-Emili-2015
EIF2B1	EIF2B4	0.078932665	Physical Interactions	Wan-Emili-2015
EIF2B2	EIF2B4	0.19061083	Physical Interactions	Wan-Emili-2015
EIF2B2	EIF2B1	0.10675983	Physical Interactions	Wan-Emili-2015
ADI1	MRI1	0.48739573	Physical Interactions	Wan-Emili-2015
NUP133	C19orf53	0.4929426	Physical Interactions	Wan-Emili-2015
EIF2B2	EIF2B4	0.2986748	Physical Interactions	Havugimana-Emili-2012
RPE	MRI1	0.59112567	Physical Interactions	Havugimana-Emili-2012
LRRC40	MRI1	0.33863264	Physical Interactions	Havugimana-Emili-2012
EIF2B2	EIF2B1	0.2345989	Physical Interactions	BIOGRID-SMALL-SCALE-STUDIES
EIF2B1	EIF2B4	0.11035627	Physical Interactions	Boldt-Roepman-2016
EIF2B2	EIF2B4	0.11035627	Physical Interactions	Boldt-Roepman-2016
EIF2B2	EIF2B1	0.122514024	Physical Interactions	Boldt-Roepman-2016
EIF2B1	EIF2B4	0.08031491	Physical Interactions	Huttlin-Harper-2017
EIF2B2	EIF2B4	0.046978123	Physical Interactions	Huttlin-Harper-2017
EIF2B2	EIF2B1	0.05582611	Physical Interactions	Huttlin-Harper-2017
KDM8	CCDC130	0.34693837	Physical Interactions	Huttlin-Harper-2017

JUP	CCDC130	0.74718094	Physical Interactions	IREF-mint
KDM8	CCDC130	0.57940745	Physical Interactions	Huttlin-Gygi-2015
JUP	CCDC130	1	Physical Interactions	Hegele-Stelzl-2012 B
JUP	CCDC130	0.183492	Physical Interactions	IREF-matrixdb
EIF2B2	EIF2B4	0.851885	Physical Interactions	IREF-huri
EIF2B2	EIF2B4	1	Physical Interactions	Fragoza-Yu-2019
EIF2B2	EIF2B4	1	Physical Interactions	Sahni-Vidal-2015
EIF2B1	MTAP	0.023497041	Physical Interactions	IREF-biogrid
EIF2B2	EIF2B1	0.01772435	Physical Interactions	IREF-biogrid
PNP	MTAP	0.039091997	Physical Interactions	IREF-biogrid
JUP	CCDC130	0.026010666	Physical Interactions	IREF-biogrid
NUP133	C19orf53	0.13340706	Physical Interactions	IREF-biogrid
EIF2B2	EIF2B1	0.024295565	Predicted	I2D-BioGRID-Yeast2Human
RDH12	MRI1	0.1441021	Predicted	I2D-BioGRID-Yeast2Human
NOB1	MRI1	0.09852965	Predicted	I2D-BioGRID-Yeast2Human
NOB1	MTAP	0.1092714	Predicted	I2D-BioGRID-Yeast2Human
EIF2B2	EIF2B1	0.33333334	Predicted	I2D-vonMering-Bork-2002-High-Yeast2Human
TOP2B	MRI1	0.56906074	Predicted	I2D-vonMering-Bork-2002-Medium-Yeast2Human
EIF2B2	EIF2B1	1	Predicted	I2D-Tarassov-PCA-Yeast2Human
MTAP	MRI1	0.57735026	Predicted	I2D-vonMering-Bork-2002-Low-Yeast2Human
MEMO1	MRI1	0.57735026	Predicted	I2D-vonMering-Bork-2002-Low-Yeast2Human
PNP	MRI1	0.57735026	Predicted	I2D-vonMering-Bork-2002-Low-Yeast2Human
YJU2	CCDC130	0.06515864	Predicted	Stuart-Kim-2003
EIF2B2	EIF2B1	0.20812462	Predicted	Stuart-Kim-2003
EIF2B2	EIF2B1	0.02023508	Predicted	I2D-IntAct-Yeast2Human
EIF2B2	EIF2B1	0.39437068	Predicted	I2D-BIND-Yeast2Human
YJU2	CCDC130	1	Shared protein domains	INTERPRO
EIF2B4	MRI1	0.14840908	Shared protein domains	INTERPRO
EIF2B1	MRI1	0.14840908	Shared protein domains	INTERPRO

EIF2B1	EIF2B4	0.14840879	Shared protein domains	INTERPRO
EIF2B2	MRI1	0.14840908	Shared protein domains	INTERPRO
EIF2B2	EIF2B4	0.14840879	Shared protein domains	INTERPRO
EIF2B2	EIF2B1	0.14840879	Shared protein domains	INTERPRO
PNP	MTAP	0.46064046	Shared protein domains	INTERPRO
YJU2	CCDC130	1	Shared protein domains	PFAM
EIF2B4	MRI1	0.33333334	Shared protein domains	PFAM
EIF2B1	MRI1	0.33333334	Shared protein domains	PFAM
EIF2B1	EIF2B4	0.33333334	Shared protein domains	PFAM
EIF2B2	MRI1	0.33333334	Shared protein domains	PFAM
EIF2B2	EIF2B4	0.33333334	Shared protein domains	PFAM
EIF2B2	EIF2B1	0.33333334	Shared protein domains	PFAM
PNP	MTAP	0.33333334	Shared protein domains	PFAM

Table S7: Biological processes characterizing the joint functions of genes in a network of interactions of C19orf53 and cis-eQTL-related genes

Function	FDR	Genes in network	Genes in genome
methionine metabolic process	0.0000082010273414672	4	12
cellular metabolic compound salvage	0.0000082010273414672	5	34
sulfur amino acid biosynthetic process	0.0000110382456167156 53	4	14
aspartate family amino acid biosynthetic process	0.000019635656934320 58	4	17
sulfur amino acid metabolic process	0.00023446820064271153	4	32
aspartate family amino acid metabolic process	0.0006032532894390611	4	42
alpha-amino acid biosynthetic process	0.0019346411271668174	4	58
cellular amino acid biosynthetic process	0.0019424727038237595	4	60
oligodendrocyte differentiation	0.030868635173738772	3	39

Table S8: Effects of rs10104 *C19orf53* on the binding of DNA to TFs

№	Ref/SNP allele[1]	TF[2]	GAIN /LOSS[3]	Motif[4]	P-Value SNP impact[5]	P-Value Ref[6]	P-Value SNP[7]
1	A/G	ELF1	gain	ELF1_disc2	0	0.187	0.0000003
2	A/G	MYC	gain	MYC_disc10	0	0.382	0.0000003
3	A/G	ETS	gain	ETS_1	0.000002	0.265	0.003
4	A/G	ELF1	gain	MA0473.1	0.0001	0.221	0.0091
5	A/G	GABPA	gain	GABPA_1	0.0005	0.432	0.003
6	A/G	ELK1	gain	ELK1_2	0.001	0.407	0.008
7	A/G	FEV	gain	FEV_1	0.001	0.259	0.025
8	A/G	Ets1	gain	MA0098.2	0.001	0.275	0.008
9	A/G	FLI1	gain	MA0475.1	0.001	0.083	0.004
10	A/G	GABPA	gain	MA0062.2	0.002	0.097	0.003
11	A/G	REST	gain	REST_disc4	0.002	0.267	0.007
12	A/G	ELF5	gain	ELF5_4	0.002	0.817	0.013
13	A/G	FLI1	gain	FLI1_1	0.002	0.822	0.010
14	A/G	EGR1	gain	EGR1_2	0.003	0.057	0.003
15	A/G	ELF5	gain	ELF5_2	0.003	0.686	0.012
16	A/G	ELK1	gain	ELK1_5	0.003	0.718	0.018
17	A/G	ETS1	gain	ETS1_2	0.003	0.074	0.002
18	A/G	ETS	gain	ETS_disc2	0.003	0.276	0.022
19	A/G	AP1	gain	AP1_disc10	0.003	0.255	0.017
20	A/G	EGR1	gain	EGR1_disc2	0.003	0.063	0.003
21	A/G	ELK1	gain	ELK1_4	0.003	0.667	0.015
22	A/G	ERF	gain	ERF_1	0.004	0.697	0.019
23	A/G	ELF4	gain	ELF4_1	0.004	0.155	0.004
24	A/G	ETV5	gain	ETV5_1	0.004	0.652	0.018
25	A/G	BHLHE40	gain	BHLHE40_disc2	0.004	0.263	0.019
26	A/G	ELK4	gain	ELK4_2	0.004	0.698	0.015
27	A/G	Erg	gain	MA0474.1	0.004	0.118	0.007
28	A/G	GABPA	gain	GABPA_3	0.004	0.725	0.014
29	A/G	SRF	gain	SRF_disc2	0.005	0.298	0.027
30	A/G	ZNF784	gain	ZNF784_1	0.006	0.539	0.019
31	A/G	ZNF589	gain	ZNF589_1	0.006	0.364	0.049
32	A/G	ERG	gain	ERG_3	0.006	0.860	0.014
33	A/G	ELK4	gain	MA0076.2	0.006	0.082	0.004
34	A/G	ZIC2	gain	ZIC2_2	0.006	0.209	0.037
35	A/G	ETV1	gain	ETV1_1	0.007	0.792	0.027
36	A/G	ELK3	gain	ELK3_2	0.007	0.695	0.021
37	A/G	ELF3	gain	ELF3_2	0.007	0.498	0.015
38	A/G	ETS1	gain	ETS1_6	0.007	0.714	0.014
39	A/G	ERG	gain	ERG_1	0.007	0.401	0.014
40	A/G	ETS1	gain	ETS1_4	0.008	0.450	0.012
41	A/G	TFAP2A	gain	TFAP2A_5	0.008	0.196	0.019
42	A/G	GATA	gain	GATA_disc3	0.008	0.253	0.012
43	A/G	ELF1	gain	ELF1_disc1	0.008	0.272	0.018

44	A/G	REST	gain	REST_disc5	0.009	0.315	0.033
45	A/G	TFAP2C	gain	ELF1_disc2	0	0.187	0.0000003
46	A/G	YY1	gain	MYC_disc10	0	0.382	0.0000003
47	A/G	STAT3	gain	ETS_1	0.000002	0.265	0.003
48	A/G	ETV4	gain	MA0473.1	0.0001	0.221	0.0091
49	A/G	ZIC3	gain	GABPA_1	0.0005	0.432	0.003
50	A/G	FLI1	gain	ELK1_2	0.001	0.407	0.008
51	A/G	E2F4	gain	FEV_1	0.001	0.259	0.025
52	A/G	BARHL1	gain	MA0098.2	0.001	0.275	0.008
53	A/G	ETV7	gain	MA0475.1	0.001	0.083	0.004
54	A/G	ELK3	gain	MA0062.2	0.002	0.097	0.003
55	A/G	ETV3	gain	REST_disc4	0.002	0.267	0.007
56	A/G	PROX1	gain	ELF5_4	0.002	0.817	0.013
57	A/G	ELK1	gain	FLI1_1	0.002	0.822	0.010
58	A/G	SRF	gain	EGR1_2	0.003	0.057	0.003
59	A/G	ELK1	gain	ELF5_2	0.003	0.686	0.012
60	A/G	TCF12	gain	ELK1_5	0.003	0.718	0.018
61	A/G	SRF	gain	ETS1_2	0.003	0.074	0.002
62	A/G	ETV2	gain	ETS_disc2	0.003	0.276	0.022
63	A/G	FEV	gain	AP1_disc10	0.003	0.255	0.017
64	A/G	BCL	gain	EGR1_disc2	0.003	0.063	0.003
65	A/G	STAT1	gain	ELK1_4	0.003	0.667	0.015
66	A/G	EHF	gain	ERF_1	0.004	0.697	0.019
67	A/G	ETS1	gain	ELF4_1	0.004	0.155	0.004
68	A/G	ELF3	gain	ETV5_1	0.004	0.652	0.018
69	A/G	EGR1	gain	BHLHE40_disc2	0.004	0.263	0.019
70	A/G	ETV4	gain	ELK4_2	0.004	0.698	0.015
71	A/G	ZBTB33	gain	MA0474.1	0.004	0.118	0.007
72	A/G	E2F	gain	GABPA_3	0.004	0.725	0.014
73	A/G	HOXA3	gain	SRF_disc2	0.005	0.298	0.027
74	A/G	EGR1	gain	ZNF784_1	0.006	0.539	0.019
75	A/G	Atoh1	gain	ZNF589_1	0.006	0.364	0.049
76	A/G	SOX10	gain	ERG_3	0.006	0.860	0.014
77	A/G	E2F4	gain	MA0076.2	0.006	0.082	0.004
78	A/G	ETS	gain	ZIC2_2	0.006	0.209	0.037
79	A/G	ELF5	gain	ETV1_1	0.007	0.792	0.027
80	A/G	TP63	loss	TP63_1	0.002	0.013	0.143
81	A/G	RBPJ	loss	RBPJ_2	0.002	0.007	0.161
82	A/G	RAD21	loss	RAD21_disc3	0.004	0.007	0.083
83	A/G	ZBTB7A	loss	ZBTB7A_disc1	0.005	0.020	0.218
84	A/G	SPZ1	loss	SPZ1_1	0.008	0.038	0.382
85	A/G	BARHL2	loss	BARHL2_4	0.009	0.043	0.437
86	A/G	GABP	loss	TP63_1	0.002	0.013	0.143
87	A/G	FEV	loss	RBPJ_2	0.002	0.007	0.161

88	A/G	NR3C1	loss	RAD21_disc3	0.004	0.007	0.083
89	A/G	ELF2	loss	ZBTB7A_disc1	0.005	0.020	0.218
	G[8]	interleukin-9-mediated signaling pathway (GO:0038113; FDR = **0.0245**); neuron fate determination (GO:0048664; FDR = **0.0375**); positive regulation of neuron differentiation (GO:0045666; FDR = **0.0105**); regulation of endothelial cell proliferation (GO:0001936; FDR = **0.0353**); negative regulation of neurogenesis (GO:0050768; FDR = **0.0424**).					
	A[9]	-					

1 – reference (Ref) / alternative (SNP) allele;
2 – TF - transcription factor;
3 – binding of TF to the reference (LOSS) / alternative (GAIN) allele;
4 – binding sites with high affinity for TF;
5 – p value statistically confirming the potential gain or loss of function of the genomic region with SNP in terms of transcription factor binding;
6 – p-value for assessing the binding of TF to the Ref allele;
7 – p-value for assessing the binding of TF to the SNP allele;
8 – biological processes pathogenetically significant for IS, in which TFs that bind to the alternative allele are jointly involved (data from the Gene Ontology resource; http://geneontology.org/);
9 – biological processes pathogenetically significant for AI, in which TFs that bind to the reference allele are jointly involved (data from the Gene Ontology resource; http://geneontology.org/)

Table S9: Effects of rs11666524 *C19orf53* on the binding of DNA to TFs

№	Ref/SNP allele [1]	TF[2]	GAIN /LOSS[3]	Motif[4]	P-Value SNP impact[5]	P-Value Ref[6]	P-Value SNP[7]
1	G/A	REST	gain	REST_disc1	0.001	0.461	0.011
2	G/A	SMC3	gain	SMC3_disc2	0.002	0.059	0.005
3	G/A	MAF	gain	MAF_1	0.003	0.099	0.011
4	G/A	SMC3	gain	SMC3_disc4	0.004	0.056	0.004
5	G/A	TCF4	gain	TCF4_1	0.005	0.507	0.022
6	G/A	RXRA	gain	RXRA_disc1	0.006	0.348	0.025
7	G/A	TCF12	gain	TCF12_disc1	0.006	0.455	0.011
8	G/A	RAD21	gain	RAD21_disc10	0.009	0.125	0.010
9	G/A	Stat4	gain	MA0518.1	0.011	0.144	0.011
10	G/A	E2F7	gain	E2F7_1	0.011	0.123	0.007
11	G/A	SREBF1	gain	SREBF1_3	0.013	0.383	0.043
12	G/A	TAL1	gain	TAL1_disc1	0.014	0.295	0.027
13	G/A	ID4	gain	ID4_1	0.020	0.307	0.026
14	G/A	GCM1	gain	GCM1_2	0.025	0.272	0.027
15	G/A	GCM1	gain	GCM1_1	0.026	0.343	0.045
16	G/A	MYF	gain	MYF_1	0.030	0.162	0.025
17	G/A	CTCF	gain	CTCF_disc6	0.031	0.309	0.041
18	G/A	CHD2	gain	CHD2_disc1	0.046	0.239	0.038
19	G/A	HNF4	gain	HNF4_disc2	0.046	0.264	0.041
20	G/A	ELF1	loss	ELF1_disc3	0.0002	0.001	0.251
21	G/A	NFATC1	loss	NFATC1_3	0.001	0.007	0.235

22	G/A	E2F1	loss	E2F1_13	0.001	0.013	0.324
23	G/A	ERF	loss	ERF_1	0.002	0.010	0.649
24	G/A	NR3C1	loss	NR3C1_6	0.003	0.014	0.205
25	G/A	ZNF784	loss	ZNF784_1	0.003	0.011	0.542
26	G/A	ZBTB33	loss	ZBTB33_disc2	0.003	0.009	0.258
27	G/A	E2F1	loss	E2F1_6	0.005	0.007	0.270
28	G/A	E2F1	loss	E2F1_18	0.005	0.019	0.359
29	G/A	E2F1	loss	E2F1_8	0.006	0.010	0.315
30	G/A	ETS	loss	ETS_disc4	0.007	0.027	0.345
31	G/A	ELK1	loss	ELK1_6	0.008	0.011	0.107
32	G/A	SRF	loss	SRF_disc2	0.009	0.043	0.300
33	G/A	AP1	loss	AP1_disc7	0.010	0.033	0.260
34	G/A	ELK4	loss	ELK4_1	0.011	0.012	0.165
35	G/A	PTF1A	loss	PTF1A_1	0.012	0.017	0.160
36	G/A	ATF3	loss	ATF3_disc3	0.012	0.005	0.076
37	G/A	SPDEF	loss	SPDEF_6	0.012	0.023	0.292
38	G/A	AR	loss	AR_6	0.016	0.046	0.629
39	G/A	TATA	loss	TATA_disc3	0.023	0.021	0.255
40	G/A	NRF1	loss	MA0506.1	0.031	0.021	0.200
41	G/A	NR2C2	loss	NR2C2_disc1	0.040	0.027	0.142
42	G/A	CENPB	loss	CENPB_1	0.041	0.048	0.262
43	G/A	FOXG1	loss	FOXG1_4	0.047	0.027	0.151
44	G/A	TCF3	loss	TCF3_6	0.047	0.019	0.078
	A[8]	astrocyte fate commitment (GO:0060018; FDR = **0.00306**); positive regulation of neuron differentiation (GO:0045666; FDR = **0.0146**).					
	G[9]	-					

1 – reference (Ref) / alternative (SNP) allele;
2 – TF - transcription factor;
3 – binding of TF to the reference (LOSS) / alternative (GAIN) allele;
4 – binding sites with high affinity for TF;
5 – p value statistically confirming the potential gain or loss of function of the genomic region with SNP in terms of transcription factor binding;
6 – p-value for assessing the binding of TF to the Ref allele;
7 – p-value for assessing the binding of TF to the SNP allele;
8 – biological processes pathogenetically significant for IS, in which TFs that bind to the SNP allele are jointly involved (data from the Gene Ontology resource; http://geneontology.org/);
9 – biological processes pathogenetically significant for IS, in which TFs that bind to reference allele are jointly involved (data from the Gene Ontology resource; http://geneontology.org/)

Table S10: Effects of rs346157 *C19orf53* on the binding of DNA to TFs

№	Ref/SNP allele[1]	TF[2]	GAIN /LOSS[3]	Motif[4]	P-Value SNP impact[5]	P-Value Ref[6]	P-Value SNP[7]
1	A/G	SRF	gain	SRF_disc2	0	0.055	<0.001
2	A/G	ZNF143	gain	ZNF143_disc4	0	0.142	<0.001
3	A/G	HDAC2	gain	HDAC2_disc4	0.0000002	0.526	<0.001
4	A/G	YY1	gain	YY1_disc5	0.000001	0.243	<0.001

5	A/G	MAZ	gain	MAZ_1	0.001	0.125	0.008
6	A/G	ELF1	gain	ELF1_disc2	0.003	0.172	0.010
7	A/G	E2F	gain	E2F_disc8	0.003	0.133	0.005
8	A/G	RARG	gain	RARG_9	0.004	0.568	0.035
9	A/G	CREB3L2	gain	CREB3L2_2	0.007	0.412	0.018
10	A/G	RAD21	gain	RAD21_disc8	0.011	0.214	0.030
11	A/G	PLAG1	gain	MA0163.1	0.012	0.107	0.019
12	A/G	REST	gain	REST_disc5	0.014	0.281	0.041
13	A/G	REST	gain	SRF_disc2	0	0.055	0
14	A/G	FOS	gain	ZNF143_disc4	0	0.142	0.0000001
15	A/G	BDP1	gain	HDAC2_disc4	0.0000002	0.526	0.00001
16	A/G	RAD21	gain	YY1_disc5	0.000001	0.243	0.0000005
17	A/G	GATA	gain	MAZ_1	0.001	0.125	0.0080
18	A/G	TFCP2	gain	ELF1_disc2	0.003	0.172	0.014
19	A/G	JUN (var.2)	gain	E2F_disc8	0.003	0.133	0.005
20	A/G	AP1	gain	RARG_9	0.004	0.568	0.035
21	A/G	STAT	gain	CREB3L2_2	0.007	0.412	0.018
22	A/G	E2F1	gain	RAD21_disc8	0.011	0.214	0.030
23	A/G	MAF	loss	MAF_2	0.0001	0.001	0.893
24	A/G	NFE2L2	loss	NFE2L2_2	0.001	0.019	0.307
25	A/G	WT1	loss	WT1_1	0.002	0.01	0.408
26	A/G	ATF4	loss	ATF4_2	0.002	0.023	0.851
27	A/G	AP1	loss	AP1_7	0.003	0.022	0.703
28	A/G	CREB1	loss	CREB1_2	0.003	0.012	0.342
29	A/G	REST	loss	REST_3	0.004	0.023	0.115
30	A/G	SP1	loss	SP1_disc3	0.004	0.02	0.443
31	A/G	EP300	loss	EP300_disc2	0.005	0.033	0.823
32	A/G	AP1	loss	AP1_10	0.006	0.031	1
33	A/G	NHLH1	loss	NHLH1_3	0.006	0.015	0.418
34	A/G	BACH2	loss	BACH2_1	0.006	0.011	0.264
35	A/G	JDP2	loss	JDP2_2	0.006	0.042	1
36	A/G	ATF2	loss	ATF2_2	0.006	0.014	0.236
37	A/G	HMGN3	loss	HMGN3_disc1	0.007	0.01	0.131
38	A/G	CREB1	loss	CREB1_9	0.007	0.02	0.392
39	A/G	PAX4	loss	PAX4_3	0.007	0.038	0.98
40	A/G	TFAP2	loss	TFAP2_disc1	0.008	0.012	0.146
41	A/G	CACBP	loss	CACBP_1	0.008	0.010	0.132
42	A/G	RHOXF1	loss	RHOXF1_3	0.008	0.028	0.531
43	A/G	RHOXF1	loss	RHOXF1_5	0.008	0.017	0.286
44	A/G	BCL	loss	BCL_disc2	0.010	0.028	0.242
45	A/G	AP1	loss	AP1_9	0.010	0.049	1
46	A/G	EP300	loss	EP300_disc1	0.010	0.006	0.096
47	A/G	ESRRA	loss	ESRRA_6	0.011	0.008	0.120
48	A/G	RXRA	loss	RXRA_disc4	0.011	0.023	0.188

49	A/G	BACH1	loss	BACH1_1	0.011	0.024	0.208
50	A/G	JUNB	loss	MA0490.1	0.012	0.024	0.208
51	A/G	PRDM1	loss	PRDM1_disc2	0.013	0.031	0.227
52	A/G	FOSL2	loss	MA0478.1	0.013	0.021	0.217
53	A/G	TAL1	loss	TAL1_disc1	0.014	0.018	0.188
54	A/G	BATF	loss	BATF_disc1	0.015	0.026	0.197
55	A/G	NHLH1	loss	MA0048.1	0.015	0.022	0.184
56	A/G	CACD	loss	CACD_1	0.015	0.028	0.202
57	A/G	HSF1	loss	HSF1_2	0.016	0.045	0.255
58	A/G	Bach1::Mafk	loss	MA0591.1	0.016	0.010	0.068
59	A/G	CREB1	loss	CREB1_6	0.018	0.030	0.386
60	A/G	FOSL1	loss	MA0477.1	0.019	0.019	0.172
61	A/G	EGR4	loss	MAF_2	0.000	0.001	0.893
62	A/G	E2F7	loss	NFE2L2_2	0.001	0.019	0.307
63	A/G	NR3C1	loss	WT1_1	0.002	0.010	0.408
64	A/G	PAX5	loss	ATF4_2	0.002	0.023	0.851
65	A/G	JUN::FOS	loss	AP1_7	0.003	0.022	0.703
66	A/G	MEF2	loss	CREB1_2	0.003	0.012	0.342
67	A/G	JUND	loss	REST_3	0.004	0.023	0.115
68	A/G	E2F1	loss	SP1_disc3	0.004	0.020	0.443
69	A/G	ZNF143	loss	EP300_disc2	0.005	0.033	0.823
70	A/G	TCF7L2	loss	AP1_10	0.006	0.031	1.000
71	A/G	E2F6	loss	NHLH1_3	0.006	0.015	0.418
72	A/G	GLI2	loss	BACH2_1	0.006	0.011	0.264
73	A/G	JDP2	loss	JDP2_2	0.006	0.042	1.000
74	A/G	CREB1	loss	ATF2_2	0.006	0.014	0.236
75	A/G	PAX5	loss	HMGN3_disc1	0.007	0.010	0.131
76	A/G	ELK1	loss	CREB1_9	0.007	0.020	0.392
77	A/G	TRIM28	loss	PAX4_3	0.007	0.038	0.980
78	A/G	IRF	loss	TFAP2_disc1	0.008	0.012	0.146
79	A/G	BHLHE40	loss	CACBP_1	0.008	0.010	0.132
80	A/G	EGR3	loss	RHOXF1_3	0.008	0.028	0.531
81	A/G	POU5F1	loss	RHOXF1_5	0.008	0.017	0.286
82	A/G	SPDEF	loss	BCL_disc2	0.010	0.028	0.242
83	A/G	Pou5f1::Sox2	loss	AP1_9	0.010	0.049	1.000
84	A/G	MAFB	loss	EP300_disc1	0.010	0.006	0.096
85	A/G	MAFK	loss	ESRRA_6	0.011	0.008	0.120

	G[8]	positive regulation of glial cell proliferation (GO:0060252; FDR = **0.0259**); cellular response to reactive oxygen species (GO:0034614; FDR = **0.0352**); response to hypoxia (GO:0001666; FDR = **0.0197**); mononuclear cell differentiation (GO:1903131; FDR = **0.0371**).
	A[9]	PERK-mediated unfolded protein response (GO:0036499; FDR = **0.015**); regulation of cellular response to heat (GO:1900034; FDR = **0.0492**); cellular response to angiotensin (GO:1904385; FDR = **0.00475**); positive regulation of neuron apoptotic process (GO:0043525; FDR = **0.00157**); positive regulation of protein binding (GO:0032092; FDR = **0.00685**);

		signal transduction by p53 class mediator (GO:0072331; FDR = **0.0071**);<br>cellular response to hypoxia (GO:0071456; FDR = **0.00167**);<br>intrinsic apoptotic signaling pathway (GO:0097193; FDR **= 0.0462**);<br>cellular response to oxidative stress (GO:0034599; FDR = **0.0154**);<br>blood vessel morphogenesis (GO:0048514; FDR = **0.00756**);<br>central nervous system development (GO:0007417; FDR = **0.0179**).

1 – reference (Ref) / alternative (SNP) allele;
2 – TF - transcription factor;
3 – binding of TF to the reference (LOSS) / alternative (GAIN) allele;
4 – binding sites with high affinity for TF;
5 – p value statistically confirming the potential gain or loss of function of the genomic region with SNP in terms of transcription factor binding;
6 – p-value for assessing the binding of TF to the Ref allele;
7 – p-value for assessing the binding of TF to the SNP allele;
8 – biological processes pathogenetically significant for IS, in which TFs that bind to the SNP allele are jointly involved (data from the Gene Ontology resource; http://geneontology.org/);
9 – biological processes pathogenetically significant for IS, in which TFs that bind to reference allele are jointly involved (data from the Gene Ontology resource; http://geneontology.org/)

Table S11: Effects of rs2277947 *C19orf53* on the binding of DNA to TFs

№	Ref/SNP allele[1]	TF[2]	GAIN /LOSS[3]	Motif[4]	P-Value SNP impact[5]	P-Value Ref[6]	P-Value SNP[7]
1	G/A	ZNF354C	gain	ZNF354C_1	0.001	0.236	0.0084
2	G/A	ESRRA	gain	ESRRA_2	0.003	0.578	0.024
3	G/A	NR1H	gain	NR1H_1	0.003	0.085	0.011
4	G/A	TAL1	gain	TAL1_5	0.004	0.222	0.018
5	G/A	ESRRA	gain	ESRRA_4	0.005	0.051	0.004
6	G/A	EN1	gain	EN1_1	0.006	0.796	0.020
7	G/A	TCF4	gain	TCF4_2	0.006	0.387	0.013
8	G/A	ZEB1	gain	ZEB1_disc1	0.006	0.326	0.016
9	G/A	FIGLA	gain	FIGLA_1	0.006	0.236	0.009
10	G/A	TCF4	gain	TCF4_1	0.007	0.724	0.040
11	G/A	MYC	gain	MYC_1	0.009	0.251	0.017
12	G/A	NR2F1	gain	NR2F1_3	0.009	0.198	0.021
13	G/A	YY2	gain	YY2_2	0.009	0.181	0.011
14	G/A	ZNF143	gain	ZNF143_disc2	0.011	0.213	0.015
15	G/A	MESP1	gain	MESP1_1	0.014	0.221	0.016
16	G/A	PAX6	gain	PAX6_2	0.018	0.469	0.041
17	G/A	SNAI2	gain	SNAI2_1	0.018	0.222	0.019
18	G/A	ZEB1	gain	ZEB1_2	0.019	0.175	0.008
19	G/A	YY1	gain	YY1_disc2	0.020	0.140	0.016
20	G/A	TCF3	gain	TCF3_1	0.021	0.183	0.029
21	G/A	RXRB	gain	RXRB_2	0.021	0.211	0.034
22	G/A	NR2F2	gain	NR2F2_2	0.021	0.358	0.037
23	G/A	TBX20	gain	TBX20_5	0.021	0.274	0.032
24	G/A	Esrrb	gain	MA0141.2	0.023	0.284	0.033

25	G/A	TFAP2A	gain	ZNF354C_1	0.001	0.236	0.008
26	G/A	NFE2	gain	ESRRA_2	0.003	0.578	0.024
27	G/A	CLOCK::ARNTL	gain	NR1H_1	0.003	0.085	0.011
28	G/A	ZBTB33	gain	TAL1_5	0.004	0.222	0.018
29	G/A	PAX5	gain	ESRRA_4	0.005	0.051	0.004
30	G/A	ZIC4	gain	EN1_1	0.006	0.796	0.020
31	G/A	TEAD2	gain	TCF4_2	0.006	0.387	0.013
32	G/A	RUNX1	gain	ZEB1_disc1	0.006	0.326	0.016
33	G/A	SPI1	loss	SPI1_disc3	0	0.0000003	0.100
34	G/A	TATA	loss	TATA_disc10	0	0.0000002	0.121
35	G/A	ELF1	loss	ELF1_disc3	0	0	0.141
36	G/A	MYC	loss	MYC_disc8	0	0	0.617
37	G/A	BCL	loss	BCL_disc10	0	0	0.136
38	G/A	BDP1	loss	BDP1_disc3	0.0001	0.007	0.094
39	G/A	CCNT2	loss	CCNT2_disc2	0.0005	0.002	0.200
40	G/A	YY1	loss	YY1_disc4	0.001	0.001	0.055
41	G/A	ZNF589	loss	ZNF589_1	0.001	0.006	0.092
42	G/A	TFAP2	loss	TFAP2_2	0.001	0.008	0.243
43	G/A	TFAP2	loss	TFAP2_3	0.002	0.014	0.375
44	G/A	HINFP	loss	HINFP_3	0.003	0.025	0.238
45	G/A	NFE2	loss	NFE2_disc4	0.003	0.015	0.206
46	G/A	TFAP2A	loss	TFAP2A_4	0.003	0.016	0.375
47	G/A	TFAP2A	loss	TFAP2A_1	0.003	0.016	0.375
48	G/A	TFAP2C	loss	TFAP2C_1	0.005	0.032	0.4324
49	G/A	RAD21	loss	RAD21_disc7	0.007	0.043	0.256
50	G/A	ATF3	loss	ATF3_disc4	0.007	0.030	0.620
51	G/A	E2F1	loss	E2F1_19	0.010	0.015	0.193
52	G/A	TFAP2A	loss	TFAP2A_2	0.013	0.036	0.263
53	G/A	REST	loss	REST_disc4	0.014	0.021	0.163
54	G/A	ZIC1	loss	ZIC1_3	0.014	0.018	0.151
55	G/A	TATA	loss	TATA_disc3	0.015	0.007	0.181
56	G/A	EGR1	loss	EGR1_disc5	0.016	0.011	0.216
57	G/A	SP1	loss	SP1_4	0.023	0.011	0.088
58	G/A	RAD21	loss	RAD21_disc4	0.026	0.039	0.451
59	G/A	ESRRA	loss	SPI1_disc3	0	0.0000003	0.100
60	G/A	KLF14	loss	TATA_disc10	0	0.0000002	0.121
61	G/A	THAP1	loss	ELF1_disc3	0	0	0.141
62	G/A	ZIC3	loss	MYC_disc8	0	0	0.617
63	G/A	EBF1	loss	BCL_disc10	0.000004	0	0.136
64	G/A	E2F1	loss	BDP1_disc3	0.0001	0.007	0.094
65	G/A	NR4A2	loss	CCNT2_disc2	0.0005	0.002	0.200

66	G/A	EGR2	loss	YY1_disc4	0.001	0.001	0.055
67	G/A	ESRRB	loss	ZNF589_1	0.001	0.006	0.092
68	G/A	MEOX2	loss	TFAP2_2	0.001	0.008	0.243
69	G/A	ESRRA	loss	TFAP2_3	0.002	0.014	0.375
70	G/A	E2F1	loss	HINFP_3	0.003	0.025	0.238
71	G/A	TFAP2C	loss	NFE2_disc4	0.003	0.015	0.206
72	G/A	SP1	loss	TFAP2A_4	0.003	0.016	0.375
73	G/A	ESRRG	loss	TFAP2A_1	0.003	0.016	0.375
74	G/A	TBX5	loss	TFAP2C_1	0.005	0.032	0.432
	A[8]	glial cell fate commitment (GO:0021781; FDR = **0.0235**); positive regulation of neuron differentiation (GO:0045666; FDR = **0.0341**); blood vessel development (GO:0001568; FDR = **0.0107**).					
	G[9]	-					

1 – reference (Ref) / alternative (SNP) allele;
2 – TF - transcription factor;
3 – binding of TF to the reference (LOSS) / alternative (GAIN) allele;
4 – binding sites with high affinity for TF;
5 – p value statistically confirming the potential gain or loss of function of the genomic region with SNP in terms of transcription factor binding;
6 – p-value for assessing the binding of TF to the Ref allele;
7 – p-value for assessing the binding of TF to the SNP allele;
8 – biological processes pathogenetically significant for IS, in which TFs that bind to the SNP allele are jointly involved (data from the Gene Ontology resource; http://geneontology.org/);
9 – biological processes pathogenetically significant for IS, in which TFs that bind to reference allele are jointly involved (data from the Gene Ontology resource; http://geneontology.org/)

Table S12: Effects of rs346158 *C19orf53* on the binding of DNA to TFs

№	Ref/SNP allele[1]	TF[2]	GAIN /LOSS[3]	Motif[4]	P-Value SNP impact[5]	P-Value Ref[6]	P-Value SNP[7]
1	T/C	YY1	gain	YY1_disc5	0.00001	0.084	0.000001
2	T/C	TEAD2	gain	TEAD2_1	0.001	0.111	0.004
3	T/C	BCL	gain	BCL_disc9	0.004	0.339	0.027
4	T/C	RUNX2	gain	RUNX2_1	0.004	0.509	0.028
5	T/C	BCL	gain	BCL_disc10	0.004	0.397	0.035
6	T/C	SPZ1	gain	SPZ1_1	0.005	0.666	0.038
7	T/C	E2F8	gain	E2F8_1	0.005	0.254	0.015
8	T/C	REST	gain	REST_disc8	0.006	0.305	0.029
9	T/C	ETS	gain	ETS_disc7	0.007	0.484	0.038
10	T/C	HINFP	gain	MA0131.1	0.008	0.117	0.008
11	T/C	E2F1	gain	E2F1_7	0.009	0.115	0.006
12	T/C	TFAP2A	gain	TFAP2A_7	0.009	0.474	0.042
13	T/C	CTCF	gain	CTCF_disc8	0.010	0.360	0.050
14	T/C	GABPA	gain	GABPA_1	0.010	0.596	0.037
15	T/C	TFAP2A	gain	TFAP2A_10	0.011	0.341	0.042
16	T/C	EGR1	gain	EGR1_disc7	0.011	0.468	0.037
17	T/C	E2F1	gain	E2F1_3	0.012	0.229	0.020
18	T/C	RUNX2	gain	RUNX2_3	0.013	0.284	0.029
19	T/C	BDP1	gain	BDP1_disc3	0.013	0.250	0.038

20	T/C	REST	gain	REST_disc4	0.014	0.330	0.042
21	T/C	ATF3	gain	ATF3_disc4	0.018	0.095	0.010
22	T/C	FLI1	gain	FLI1_1	0.020	0.462	0.037
23	T/C	ETV4	gain	ETV4_2	0.020	0.812	0.042
24	T/C	ELF4	gain	ELF4_1	0.023	0.393	0.044
25	T/C	ETV5	gain	YY1_disc5	0.00001	0.084	0.000001
26	T/C	TAL1	gain	TEAD2_1	0.001	0.111	0.004
27	T/C	ELK1	gain	BCL_disc9	0.004	0.339	0.027
28	T/C	CREB	gain	RUNX2_1	0.004	0.509	0.028
29	T/C	TAL1::GATA1	loss	MA0140.2	0.0004	0.014	0.328
30	T/C	NANOG	loss	NANOG_disc1	0.001	0.010	0.634
31	T/C	SMAD	loss	SMAD_2	0.001	0.007	0.232
32	T/C	POU	loss	POU_2	0.002	0.013	0.564
33	T/C	RFX2	loss	RFX2_2	0.003	0.009	0.104
34	T/C	HAND1	loss	HAND1_2	0.003	0.008	0.429
35	T/C	HAND1	loss	HAND1_1	0.003	0.014	0.614
36	T/C	BCL	loss	BCL_disc5	0.005	0.014	0.251
37	T/C	RXRA	loss	RXRA_disc5	0.005	0.015	0.201
38	T/C	POU2F1	loss	POU2F1_7	0.006	0.005	0.081
39	T/C	SMAD3	loss	SMAD3_2	0.006	0.034	0.527
40	T/C	Atoh1	loss	MA0461.1	0.007	0.008	0.090
41	T/C	POU2F2	loss	POU2F2_disc1	0.007	0.016	0.214
42	T/C	TGIF2LX	loss	TGIF2LX_1	0.008	0.037	0.727
43	T/C	Hand1::Tcfe2a	loss	MA0092.1	0.009	0.014	0.244
44	T/C	POU5F1	loss	POU5F1_2	0.011	0.044	0.515
45	T/C	TAL1	loss	TAL1_disc1	0.012	0.014	0.165
46	T/C	NHLH1	loss	NHLH1_4	0.012	0.027	0.508
47	T/C	POU2F1	loss	POU2F1_9	0.012	0.014	0.209
48	T/C	CTCFL	loss	CTCFL_disc1	0.017	0.013	0.1718
49	T/C	Pou5f1::Sox2	loss	MA0142.1	0.017	0.035	0.226
50	T/C	POU5F1	loss	POU5F1_3	0.017	0.033	0.218
51	T/C	POU2F3	loss	POU2F3_1	0.020	0.024	0.178
52	T/C	POU3F4	loss	POU3F4_2	0.024	0.034	0.275
53	T/C	POU3F2	loss	POU3F2_6	0.025	0.032	0.219
54	T/C	POU2F2	loss	POU2F2_1	0.025	0.024	0.143
55	T/C	EGR1	loss	MA0140.2	0.0004	0.014	0.328
56	T/C	POU2F1	loss	NANOG_disc1	0.001	0.010	0.634
57	T/C	ATF1	loss	SMAD_2	0.001	0.007	0.232
58	T/C	ZBTB14	loss	POU_2	0.002	0.013	0.564
59	T/C	OTX	loss	RFX2_2	0.003	0.009	0.104
60	T/C	PAX5	loss	HAND1_2	0.003	0.008	0.429
61	T/C	NANOG	loss	HAND1_1	0.003	0.014	0.614

	C[8]	-
	T[9]	-

1 – reference (Ref) / alternative (SNP) allele;
2 – TF - transcription factor;
3 – binding of TF to the reference (LOSS) / alternative (GAIN) allele;
4 – binding sites with high affinity for TF;
5 – p value statistically confirming the potential gain or loss of function of the genomic region with SNP in terms of transcription factor binding;
6 – p-value for assessing the binding of TF to the Ref allele;
7 – p-value for assessing the binding of TF to the SNP allele;
8 – biological processes pathogenetically significant for IS, in which TFs that bind to the SNP allele are jointly involved (data from the Gene Ontology resource; http://geneontology.org/);
9 – biological processes pathogenetically significant for IS, in which TFs that bind to reference allele are jointly involved (data from the Gene Ontology resource; http://geneontology.org/)

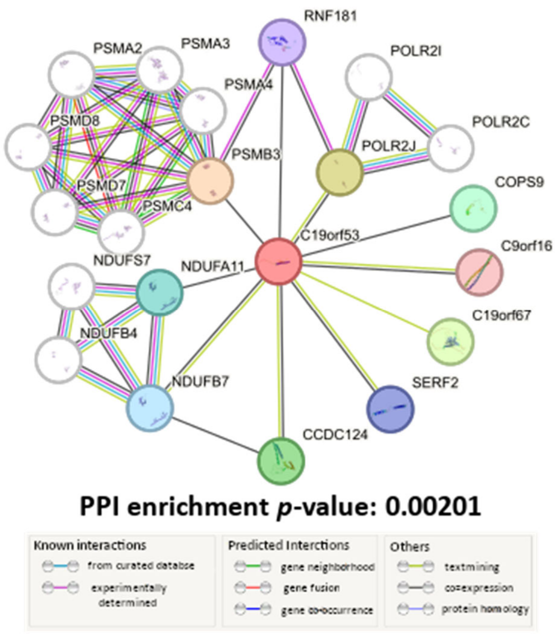

Figure S3: Predicted Interaction Partners of *C19orf53*

Table S13: Main functional characteristics of predicted functional partners of *C19orf53*

Protein	Functions	*						Score
		1	2	3	4	5	6	
POLR2J	DNA-directed RNA polymerase II subunit RPB11-a; DNA-dependent RNA polymerase catalyzes the transcription of DNA into RNA using the four ribonucleoside triphosphates as substrates. Component of RNA polymerase II which synthesizes mRNA precursors and many functional non-coding RNAs. Pol II is the central component of the basal RNA polymerase II transcription machinery. It is composed of mobile elements that move relative to each other.			•			•	0.626

	RPB11 is part of the core element with the central large cleft (By similarity).							
C19orf67	UPF0575 protein C19orf67; Chromosome 19 open reading frame 67; Belongs to the UPF0575 family.			•			•	0.584
CCDC124	Coiled-coil domain-containing protein 124; Required for proper progression of late cytokinetic stages. Belongs to the CCDC124 family.						•	0.584
COPS9	COP9 signalosome complex subunit 9; [Isoform 1]: Component of the COP9 signalosome complex (CSN), a complex involved in various cellular and developmental processes. The CSN complex is an essential regulator of the ubiquitin (Ubl) conjugation pathway by mediating the deneddylation of the cullin subunits of SCF-type E3 ligase complexes, leading to decrease the Ubl ligase activity of SCF-type complexes such as SCF, CSA or DDB2. The complex is also involved in phosphorylation of p53/TP53, c-jun/JUN, IkappaBalpha/NFKBIA, ITPK1 and IRF8/ICSBP, possibly via its association with CK2 and PKD kinases. CSN-dependent phosphorylation of TP53 and JUN promotes and protects degradation by the Ubl system, respectively. Plays a role in cell proliferation.			•			•	0.581
NDUFA11	NADH dehydrogenase [ubiquinone] 1 alpha subcomplex subunit 11; Accessory subunit of the mitochondrial membrane respiratory chain NADH dehydrogenase (Complex I), that is believed not to be involved in catalysis. Complex I functions in the transfer of electrons from NADH to the respiratory chain. The immediate electron acceptor for the enzyme is believed to be ubiquinone.			•			•	0.538
NDUFB7	NADH dehydrogenase [ubiquinone] 1 beta subcomplex subunit 7; Accessory subunit of the mitochondrial membrane respiratory chain NADH dehydrogenase (Complex I), that is believed not to be involved in catalysis. Complex I functions in the transfer of electrons from NADH to the respiratory chain. The immediate electron acceptor for the enzyme is believed to be ubiquinone.			•			•	0.528
SERF2	Small EDRK-rich factor 2; Positive regulator of amyloid protein aggregation and proteotoxicity. Induces conformational changes in amyloid proteins, such as HTT, driving them into compact formations preceding the formation of aggregates. Belongs to the SERF family.			•			•	0.500
RNF181	E3 ubiquitin-protein ligase RNF181; E3 ubiquitin-protein ligase which accepts ubiquitin from an E2 ubiquitin-conjugating enzyme in the form of a thioester and then directly transfers the ubiquitin to targeted substrates.			•			•	0.480
C9orf16	UPF0184 protein C9orf16; Chromosome 9 open reading frame 16.			•			•	0.479

Data from the STRING database is shown.

* - Proven interaction mechanisms:<br>1 –Neighborhood; 2 – Cooccurence; 3 – Coexpression; 4 – Experiments; 5 – Databases; 6 - Textmining

Table S14: Functional enrichments of *C19orf53* network

№	Term ID	Term Description	Observed Gene Count	Background Gene Count	Strength	FDR
	Biological processes (Gene Ontology)					
1	GO:0032981	Mitochondrial respiratory chain complex I assembly	4	61	1.79	0.0032
2	GO:0006120	Mitochondrial electron transport, NADH to ubiquinone	3	46	1.79	0.0121
3	GO:0042776	Proton motive force-driven mitochondrial ATP synthesis	4	64	1.77	0.0032
4	GO:0043161	Proteasome-mediated ubiquitin-dependent protein catabolic process	7	347	1.28	0.00083
5	GO:0034654	Nucleobase-containing compound biosynthetic process	8	913	0.91	0.0038
6	GO:0006807	Nitrogen compound metabolic process	16	6643	0.35	0.0354
Data from the STRING database is shown.						
